# Nonlinear tumor evolution from dysplastic nodules to hepatocellular carcinoma

**DOI:** 10.18632/oncotarget.10502

**Published:** 2016-07-09

**Authors:** Je-Gun Joung, Sang Yun Ha, Joon Seol Bae, Jae-Yong Nam, Geum-Youn Gwak, Hae-Ock Lee, Dae-Soon Son, Cheol-Keun Park, Woong-Yang Park

**Affiliations:** ^1^ Samsung Genome Institute, Samsung Medical Center, Sungkyunkwan University School of Medicine, Seoul, Korea; ^2^ Samsung Advanced Institute for Health Sciences and Technology, Samsung Medical Center, Sungkyunkwan University School of Medicine, Seoul, Korea; ^3^ Samsung Biomedical Research Institute, Samsung Medical Center, Sungkyunkwan University School of Medicine, Seoul, Korea; ^4^ Departments of Molecular Cell Biology, Samsung Medical Center, Sungkyunkwan University School of Medicine, Seoul, Korea; ^5^ Departments of Pathology, Samsung Medical Center, Sungkyunkwan University School of Medicine, Seoul, Korea; ^6^ Departments of Internal Medicine, Samsung Medical Center, Sungkyunkwan University School of Medicine, Seoul, Korea

**Keywords:** hepatocellular carcinoma, dysplastic nodules, whole-exome sequencing, single nucleotide variation, copy number variation

## Abstract

Dysplastic nodules are premalignant neoplastic nodules found in explanted livers with cirrhosis. Genetic signatures of premalignant dysplastic nodules (DNs) with concurrent hepatocellular carcinoma (HCC) may provide an insight in the molecular evolution of hepatocellular carcinogenesis. We analyzed four patients with multifocal nodular lesions and cirrhotic background by whole-exome sequencing (WES). The genomic profiles of somatic single nucleotide variations (SNV) and copy number variations (CNV) in DNs were compared to those of HCCs. The number and variant allele frequency of somatic SNVs of DNs and HCCs in each patient was identical along the progression of pathological grade. The somatic SNVs in DNs showed little conservation in HCC. Additionally, CNVs showed no conservation. Phylogenetic analysis based on SNVs and copy number profiles indicated a nonlinear segregation pattern, implying independent development of DNs and HCC in each patient. Thus, somatic mutations in DNs may be developed separately from other malignant nodules in the same liver, suggesting a nonlinear model for hepatocarcinogenesis from DNs to HCC.

## INTRODUCTION

Hepatocellular carcinoma (HCC) develops in patients with chronic liver diseases [1, 2]. Chronic hepatitis and cirrhosis make up the major premalignant conditions in most HCCs [3, 4]. Premalignant lesions may be caused by viral infection, environmental chemical carcinogens, fungal toxins, as well as genetic diseases. Numerous genetic alterations initiated by chronic hepatocyte destruction and regeneration can result in neoplastic growth. Pathologically premalignant lesions of HCCs have been designated as dysplastic nodules by the International Working Party. Dysplastic nodules, which usually occur in cirrhotic livers, are further classified into low-grade dysplastic nodules (LGDNs) and high-grade dysplastic nodules (HGDNs) depending on the degree of cytological or architectural abnormalities on histological examination [5].

HCCs are multifocal in the liver in 75% of cases at diagnosis [1]. In imaging studies, various stages of the tumor and small dysplastic nodules are often detected together. Dysplastic nodules may develop into early HCC, which may further develop into progressed HCC. Progressed HCCs have poor prognosis because of a high recurrence rate. Conversely, early HCCs can be treated by curative procedures such as resection and percutaneous ablation and have a better prognosis.

Clarifying the molecular mechanisms of early hepatocarcinogenesis is an important step for the identification of novel therapeutic targets against HCC development. Chuma et al [6] reported that HSP70 could be a sensitive marker for differential diagnosis of early HCC from dysplastic nodules. A recent report demonstrated that cyclase-associated protein-2 was overexpressed in early and progressed HCCs but was absent or focally expressed in all cases of dysplastic nodules [7]. It is widely accepted that HGDNs are one of the most important precursor lesions of HCC with a cirrhotic background. A recent study presented that the frequency of telomerase reverse transcriptase promoter mutations rapidly increased during the different transformation steps of premalignant lesions into HCC [8]. Furthermore, methylation changes were gradually increased along the hepatitis B virus (HBV)-related multistep hepatocarcinogenesis [9]. However, the genetic profile of dysplastic nodules is unclear. The study of genetic alterations in dysplastic nodules may allow for the discovery of crucial genetic events associated with transformation of premalignant lesions into HCC.

The advent of next generation sequencing technologies enabled us to generate accurate genomic profiles including mutations, copy number variations (CNVs), and other alterations [10]. Recently, exome capture sequencing revealed many somatic mutations associated with HBV-related HCC [11, 12]. This high-resolution genomic information provides strong clues for the targeted therapy.

To investigate the genetic aberrations associated with the transformation of premalignant lesions into HCC on a cirrhotic background, we performed comprehensive whole-exome sequencing (WES) in four cases with LGDNs, HGDNs, and HCCs. Our investigation indicates that human hepatocarcinogenesis is a multistep process accompanied by a stepwise increase in high confidence mutations and degree of CNVs from LGDN and HGDN to HCC.

## RESULTS

### Somatic SNVs landscape of dysplastic nodules

**Figure 1:**
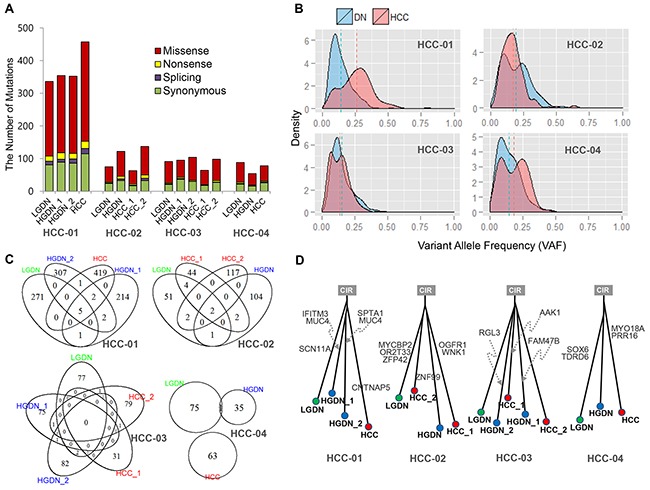
Mutations identified in dysplastic nodules (DNs) and Hepatocellular Carcinoma (HCC) **A**. The number of mutations detected; **B**. Comparison of variant allele frequency (VAF) distribution between DNs and HCC; **C**. Overlap of mutations between DNs and HCC; **D**. The phylogenetic trees inferred from the mutation profiles.

We analyzed 10 dysplastic nodules from four multinodular hepatocellular carcinoma cases together with HCC lesions and matched cirrhotic background tissues by WES (Supplementary Figure S1). The quality of WES from FFPE tissues was ensured to get mean target depths with 106.2±30.3 (Supplementary Table S1 and Supplementary Figure S2). Within each patient, the SNVs detected by default filters showed no significant difference in numbers and variant types between DN and HCC (Figure 1A). To validate the high-confidence SNVs, we performed Sanger sequencing for fifteen SNVs (Supplementary Table S2). In exonic regions, we identified 132.9±98.5 somatic single nucleotide variants (SNVs) per DN, and 128.7±147.1 SNVs per nodule for HCC. Fifty one out of 772 (6.6%) SNVs in HCC were previously reported in the COSMIC or TCGA databases (Supplementary Table S3). The total number of somatic variants in premalignant DN was similar to that of HCC. In addition, the fraction of publicly reported SNVs of DN was not different from HCC.

The overall patterns of somatic variants from DN and HCC were similar to each other. The allele fraction of somatic SNVs provides information regarding clonality and heterogeneity. Even though somatic SNVs of DN from the patient, HCC-01 and HCC-04, were distributed to the lower fraction in variant allele frequency, there was no significant difference between the variant allele frequency in DN and HCC (Figure 1B).

Interestingly, somatic SNVs in DN rarely overlapped with variants in HCC in all cases (Figure 1C). Eleven genes concordantly detected between DNs and HCC are shown in Supplementary Figure S3. In the patient HCC-04, we found only one SNV common to HCC and DN. Thus, we could not find common driver mutations in each patient based on somatic SNVs. Moreover, phylogenetic analysis of DNs and HCCs using somatic SNVs in each patient showed independent growth with few driving mutations in common (Figure 1D).

### Molecular signatures for tumor evolution in dysplastic nodules

**Figure 2:**
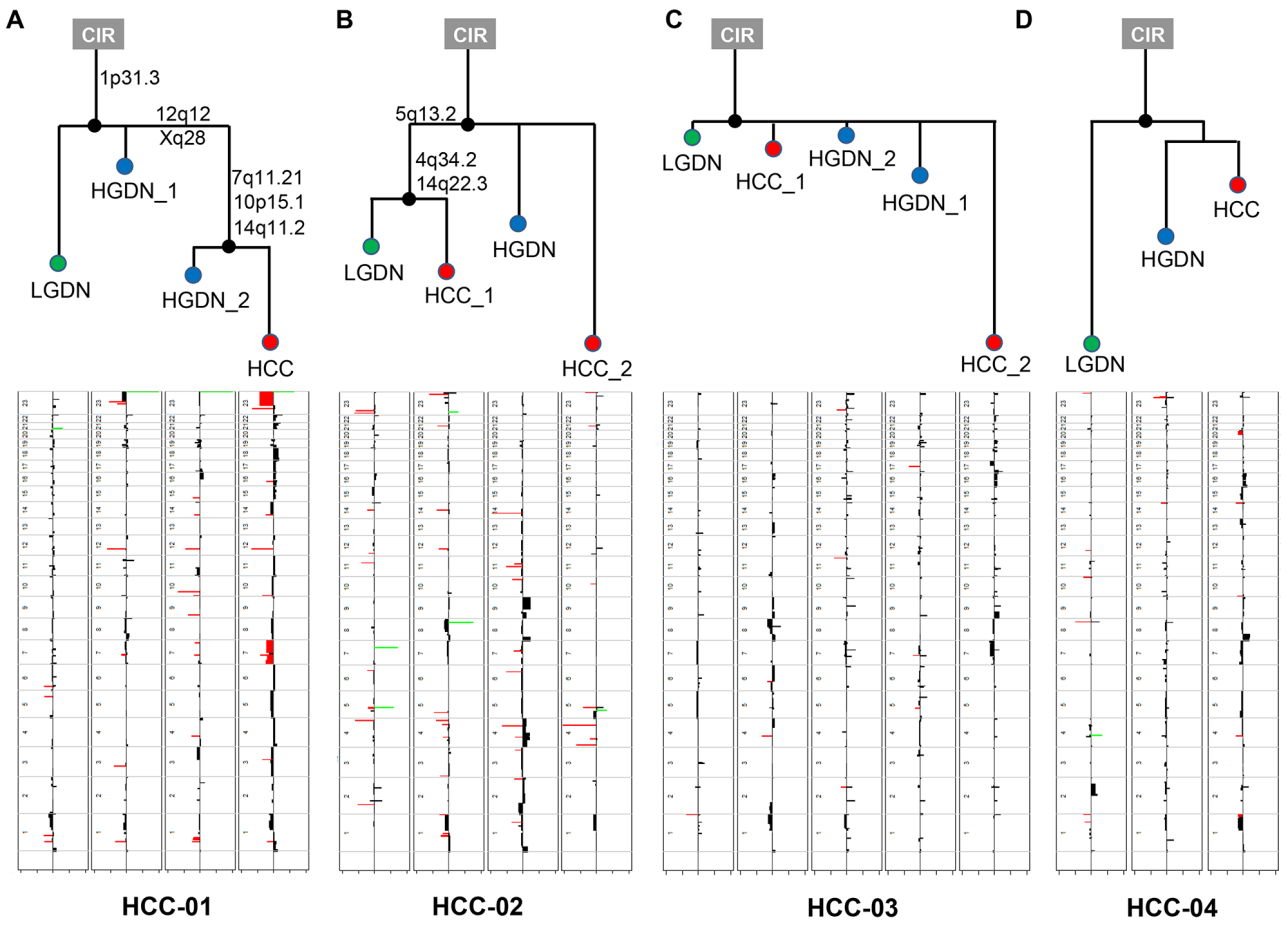
The phylogenetic trees inferred from copy number profiles of four HCC patients In the copy number profile, red and green colored bars indicate amplification and deletion, respectively.

Copy number amplification and deletion can provide evidence for tumor evolution in premalignant lesions. We detected 70 CNVs (58 amplifications and 12 deletions) in 10 DNs of four patients at the level of ≥3 or ≤0.5 copies. Six (8.5%) CNV regions overlapped with 53 previously reported CNVs loci by GISTIC [13] analysis of 208 primary HCCs in the TCGA database (Supplementary Table S4). Each nodule had about 7.0±4.5 alterations (5.8 amplifications and 1.2 deletions) on an average. In each patient, all DN and HCC regions shared a similar CNV pattern, with increasing number and area of CNVs in HGDN and HCC compared to those of LGDN. The loci and patterns of CNVs in HGDNs and HCC were quite similar within a patient (Figure 2). The degree and number of CNVs in the patient HCC-01, showed a stepwise increase with the progression of pathology.

Phylogenetic patterns of lesions in the four patients were investigated by copy number profiles using multistate discrete-characters parsimony. In HCC-01, copy number changes of the trunk nodes were shared by LGDNs, HGDNs, and HCCs. The gains on the chromosome arm 1p31.3 marked an early event commonly found in all samples. Changes in three loci, 7q11.21, 10p15.1, and 14q11.2, were preceded by progression from HGDN to HCC. Several amplification/deletion regions overlapping among LGDNs, HGDNs, and HCCs were also found in regions frequently enriched in other cancers. Especially in the HCC samples of HCC-02, relatively large regions in chromosome 7 and chromosome X were affected. In HCC-02, cirrhosis was segregated by 5q13.2, 4q34.2 and 14q22.3 into LGDN and HCCs without any changes in HGDN. This suggests that HGDNs in this case may involve another evolutionary pathway not listed in the LGDNs. Therefore, the overall pattern of the phylogenetic trees or tumor evolution suggest a nonlinear pattern in HCC and/or DN in each nodule.

## DISCUSSION

The complex process of tumor evolution ultimately involves optimization and integration of multi-level cellular alterations to achieve clonal expansion [14]. Clonal evolution involves the interplay of driver mutations with other neutral changes as well as changes in the microenvironment. Accumulation of genome data on paired primary and metastatic tumors demonstrated the evolution of clonal expansion in the tumor [15, 16]. Although most mutational processes are random, recurrent and adaptive mutations select the clones to be expanded. Increasing evidence indicates a multistep process in human hepatocarcinogenesis. A recent report demonstrated that chromosomal instability, telomere shortening, and inactivation of p21^WAF1/CIP1^ check point function occurred in LGDNs as well as in HGDNs, and their cooperation was considered critical for malignant transformation during HBV-associated multistep hepatocarcinogenesis [17]. A stepwise increase in allelic losses [18] and methylation of CpG islands of genes [9] is observed from cirrhosis through dysplastic nodules to HCC. Recently a “big-bang” model for clonal expansion of colorectal cancer has been proposed on the basis of genomic profiling of 15 colorectal cancer patients [19]. It proposed that dominant mutations in the early phase of colorectal cancer development are pervasive, whereas later alterations are localized in progressively smaller tumor subpopulations. This model does not support the view of clonal evolution where sequential mutations result in fitter clones that dominate the population. Multi-focal nodules in the liver may reflect traces of initial events, which are not further developed by the types of mutation or constrained micro-environments.

We investigated genetic aberrations associated with the transformation of premalignant lesions into HCC by WES. We analyzed LGDH, HGDN, and HCC samples from the same patients to show their spectral features and molecular alterations. However, we could not find significant changes in the number and frequency of somatic point mutations in different types of hepatic nodules. CNV in DN and HCC was not similar, and the most of the DNs did not resemble HCCs at the molecular level. The great variety in genetic aberrations common to both DNs and HCCs were not sufficient to support the view that different genetic mechanisms may entrain premalignant hepatocytes to transform into malignant cancer. In a previous study that analyzed chromosomal gains and losses using comparative genomic hybridization, the frequency and pattern of genetic alterations in dysplastic nodules resembled the alterations found in HCCs more than in liver cell adenomas and focal nodular hyperplasia [20]. We had two cases with conserved CNVs in some DNs and HCCs, but this was not observed in all cases and all nodules.

In order to obtain concrete results of the genomic alterations, several technical and analytical issues are raised. DNA derived from low quality FFPEs can be highly fragmented so that sequence artifacts may occur and they can affect the mutation analysis. Previous report demonstrated that low quality FFPEs exhibit particularly high enrichment of C:G > T:A substitutions as a consequence of cytosine deamination [21, 22]. Our sequence samples showed high quality in terms of DNA, library and mapping rate (Supplementary Table S1 and Supplementary Figure S4). Alterations of several tumor suppressor genes such as ARID1A, SETD2, NOTCH2 and ACVR1B were also detected from short insertion and deletion analysis (Supplementary Table S5). Further examination of these mutations is needed to know how they are involved in the initiation or progression of tumorigenesis in HCC. Recently, comprehensive analysis of genomic alterations in HCCs has been reported [23]. Interestingly 22% of HBV-positive samples had the HBV genome integration in the TERT locus. In order to observe this status in our samples, we performed the analysis for HBV genome integration for three HBV-positive patients. Paired-end reads were mapped to the HBV viral and human genomes. HBV virus integrations were detected in three HBV-positive cases. Especially HCC-03 case had HBV integration in TERT (Supplementary Table S6). During HCC development, the role of hepatitis B virus (HBV) integration needed to be elucidated with large cohort of DNs and HCCs. Human hepatocarcinogenesis is a multistep process accompanied by a stepwise pattern of pathological changes from LGDNs and HGDNs to HCC. The results of phylogenetic patterns inferred from SNVs and CNVs suggest that the transformation of LGDNs and HGDNs to HCC in human hepatocarcinegenesis may be not a linear process through the accumulation of genomic alterations involving early events. We propose a nonlinear evolution of DNs to HCC where, after the initial genetic alteration in separate regions of the liver, transformed cells in certain region(s) start to grow predominantly as a single expansion. The other part of the nodules persists in the liver without malignant transformation into HCC. Although new private mutations will be continuously generated by replication errors in all nodules, only the early events will pervade to form the tumor mass. Our findings indicate possible new lines of research for a “big-bang” model of HCC development.

## MATERIALS AND METHODS

### Patients and sample acquisition

Four patients suffering from chronic viral cirrhosis were subjected to liver transplantation between December 2010 and March 2012 at Samsung Medical Center, Seoul, Korea. None of the patients had received treatment for HCC before the transplantation procedure.

Freshly excised livers were serially sectioned at 3 to 4 mm intervals and examined by a pathologist for presence of nodular lesions. Bulging nodules of at least 5 mm in diameter or lesions that were macroscopically different in color from the surrounding liver, were fixed in 10% neutral formalin and embedded in paraffin. HCC, LGDN, and HGDN were confirmed histologically according to the guidelines of the International Working Party [5] (Figure 1). Every patient had each of LGDN, HGDN, and HCC lesions in their liver. The dysplastic nodules ranged from 0.8 to 1.6 cm in diameter, with a mean diameter of 1.1 cm. The size of HCCs ranged from 0.9 to 1.9 cm (mean, 1.25 cm). All four patients were men, with ages ranging from 53 to 67 years (mean, 59.5). Three patients had histologically proven cirrhosis related to HBV infection and one patient had hepatitis C virus infection (Supplementary Table S7).

Fresh frozen tissues from five other chemotherapy-naïve patients were used for validation of CNV. Informed consent was obtained from each patient included in the study. This study was approved by the institutional review board (IRB) of Samsung Medical Center (IRB approval no.: SMC2013-12-131).

### Genomic DNA preparation from FFPE tissues for WES

Ten formalin fixed paraffin embedded (FFPE) sections of 10 μm from each lesion were stained with eosin. The eosin-stained tissues were microdissected under a stereomicroscope. Genomic DNA was extracted from the FFPE tissues using the Maxwell 16 CSC DNA FFPE kit with the Maxwell MDx automated instrument (Promega, Madison, WI, USA). The degradation of FFPE tissues was measured by the 2200 TapeStation Instrument (Agilent Technologies, Santa Clara, CA, USA) according to the manufacturer's instructions.

Genomic DNA from 12 nodules and their corresponding cirrhotic tissues was sheared by the Covaris S220 (Covaris, Woburn, MA, USA) and used for the construction of a library using SureSelect XT Human All Exon v5 and SureSelect XT reagent kit, HSQ (Agilent Technologies) according to the manufacturer's protocol. This kit is designed to enrich 335,756 exons of 21,058 genes, covering ~71 Mb of the human genome. After the enriched exome libraries were multiplexed, the libraries were sequenced on HiSeq 2500 sequencing platform (Illumina, San Diego, CA, USA). Briefly, a paired-end DNA sequencing library was prepared by genomic DNA shearing, end-repair, A-tailing, paired-end adaptor ligation, and amplification. After hybridization of the library with bait sequences for 16 hr, the captured library was purified and amplified with an index barcode tag, and the library quality and quantity were measured. Sequencing of the exome library was carried out using the 100bp paired-end mode of the TruSeq Rapid PE Cluster kit and TruSeq Rapid SBS kit (Illumina).

### Bioinformatics analyses

Sequencing reads were aligned to the UCSC hg19 reference genome using Burrows-Wheeler Aligner [24], version 0.6.2 with default settings. PCR duplications are marked by Picard-tools-1.8 (http://picard.sourceforge.net/), followed by data cleanup with GATK-2.2.9 [25]. Point mutations were then identified by the MuTect tool (https://github.com/broadinstitute/mutect) using all the 12 nodules and their corresponding cirrhotic tissues (Supplementary Figure S1). Perl script and Annovar were used to annotate the variants and search the known somatic mutations (around 1.3 million) using COSMIC v.64 (http://cancer.sanger.ac.uk/) and TCGA. High confidence mutations were selected with a threshold read depth of ≥ 20, a variant allele frequency of ≥ 20%, and sequencing quality check. We checked the sequencing quality by visualizing mutations in genome regions using the Integrative Genome Viewer tool (http://www.broadinstitute.org/igv/). Among numerous CNV callers, EXCAVATOR showed the best performance in detect CNVs [26] [27]. In order to assess a stepwise increasing pattern of mutations and CNV, we obtained *P* values from 10,000 permutations under increasing alternatives [28]. Their significance was then tested with rates of high confidence mutations and the number of CNVs per chromosome, respectively. Phylogenetic analysis was performed based on copy number profiles. We generated the binary sequence by binning copy number regions at a bin size of 100,000 bp. The phylogenetic tree was then analyzed by Wagner parsimony method [29].

## SUPPLEMENTARY MATERIALS FIGURES










