# Supplementary material for: Nonlinear tumor evolution from dysplastic nodules to hepatocellular carcinoma
Source: Oncotarget. 2016 Jul 9;8(2):2076–82. doi: 10.18632/oncotarget.10502 (PMC5356781; doi:10.18632/oncotarget.10502)
Supplement: Supplementary file 1 [file oncotarget-08-2076-s001.pdf]

Nonlinear tumor evolution from dysplastic nodules to hepatocellular carcinoma

SUPPLEMENTARY FIGURES AND TABLES

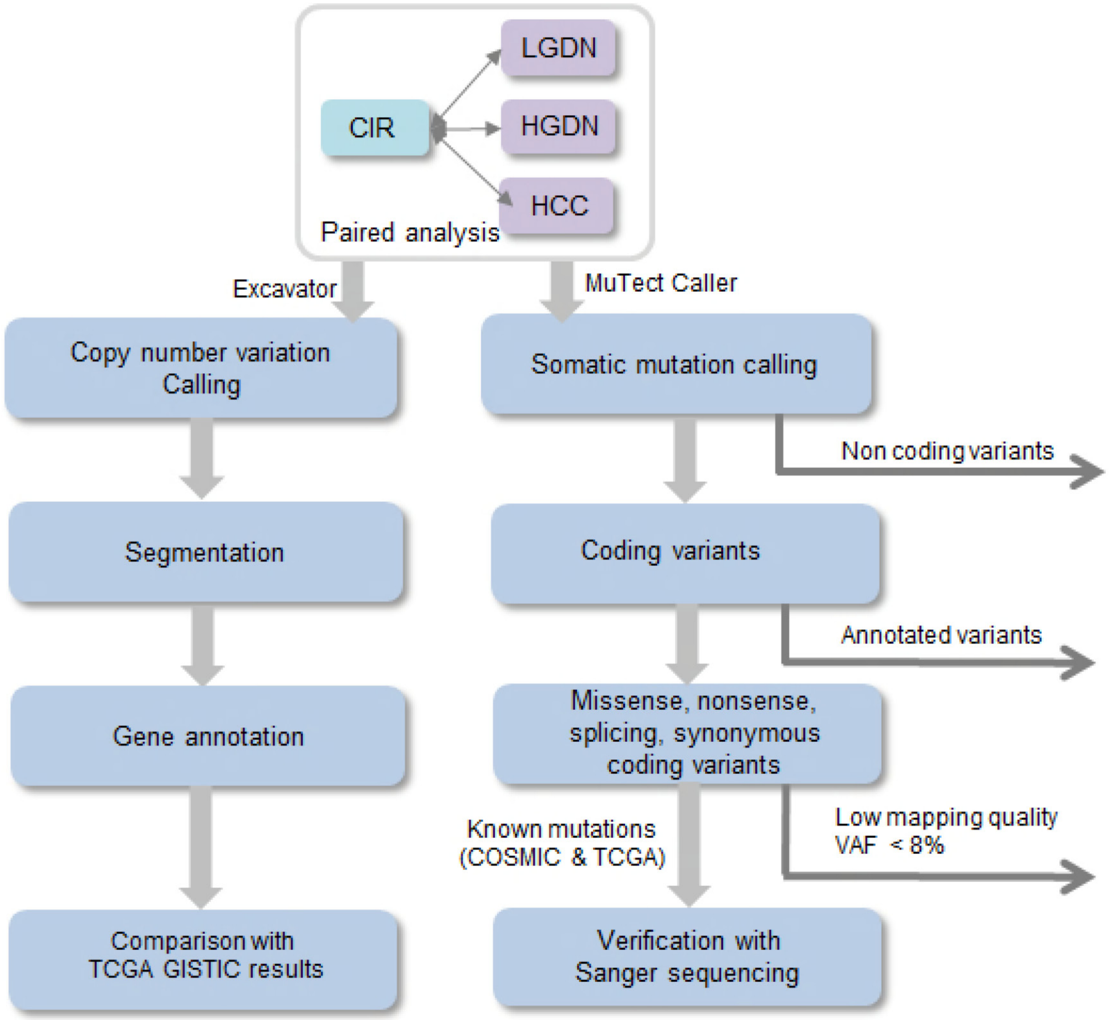

Supplementary Figure S1: Whole-exome sequencing analysis procedure.

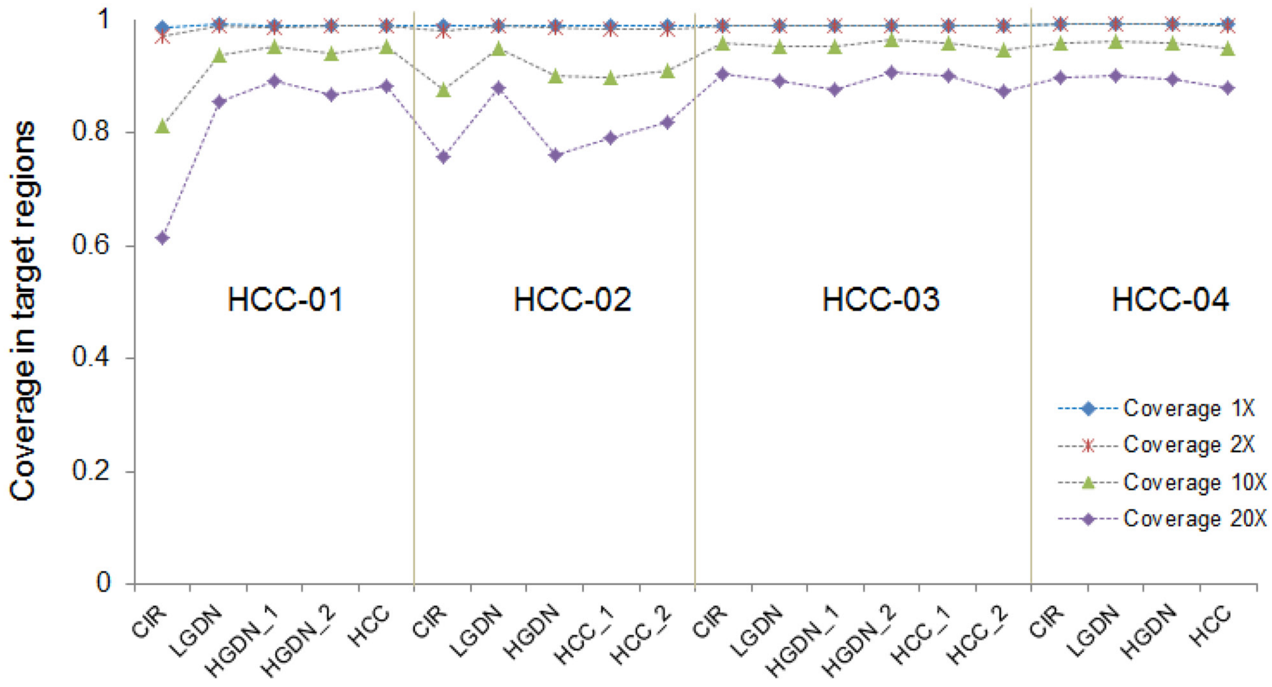

Supplementary Figure S2: Mapping statistics of sequencing reads.

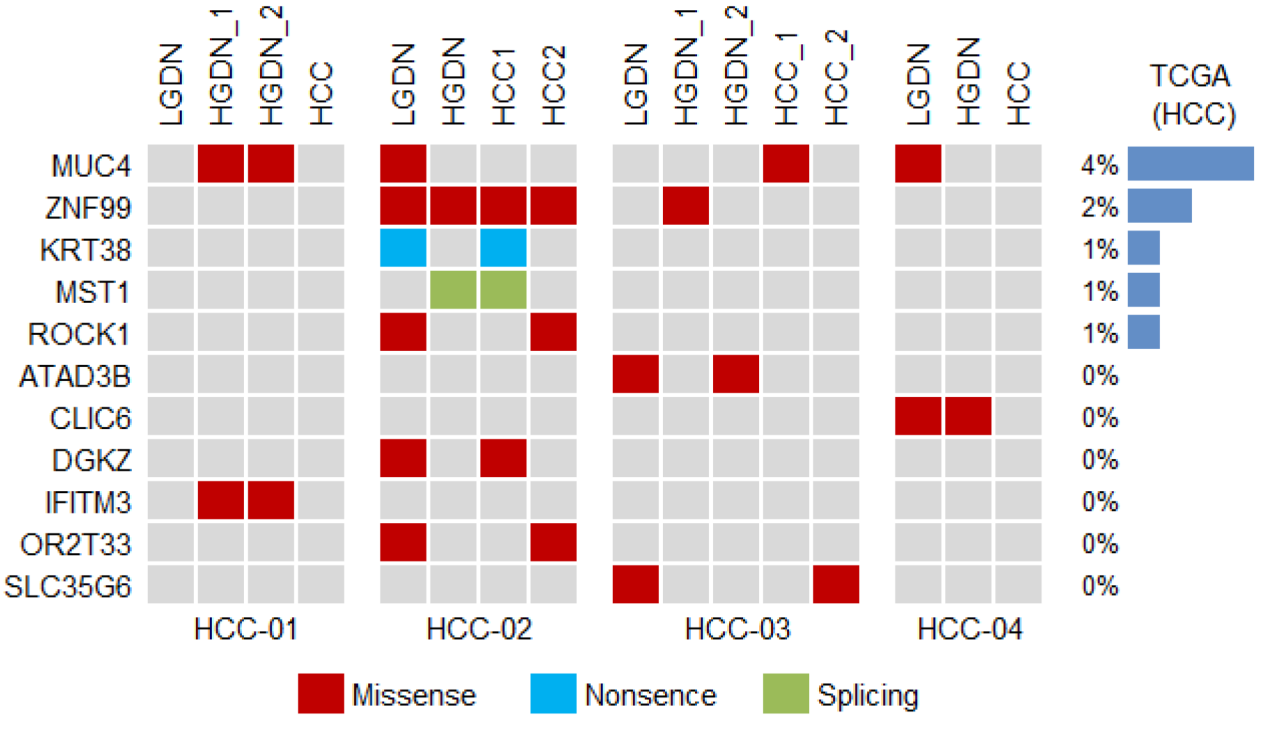

Supplementary Figure S3: Heatmap of genes mutated in nodules and HCCs.

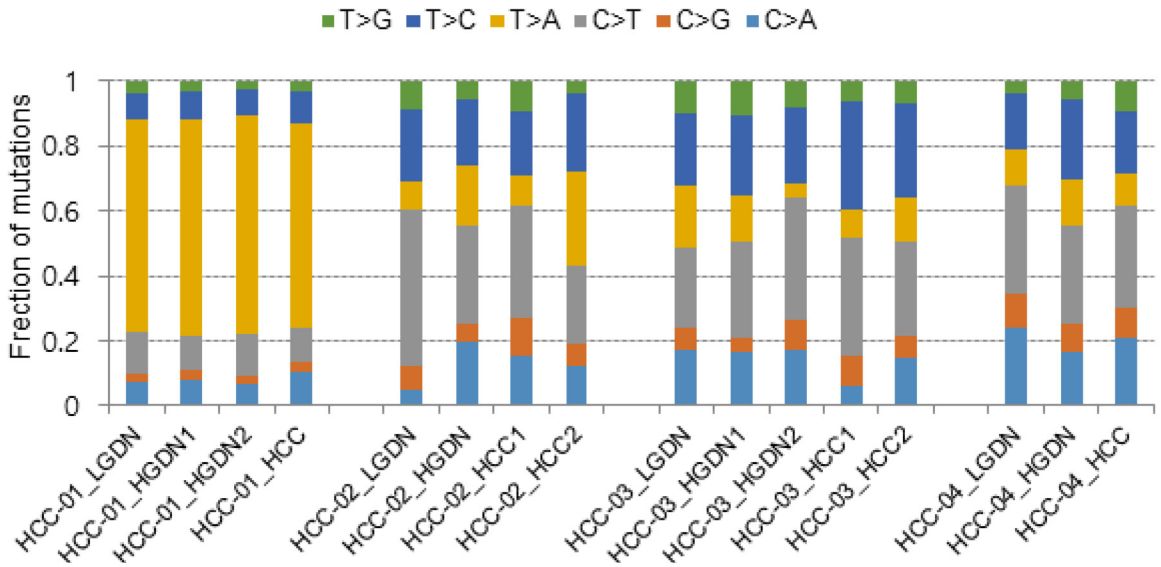

Supplementary Figure S4: SNV spectrum of each nodule and HCC sample.

Supplementary Table S1: Summary of whole exome sequencing.

See Supplementary File 1

Supplementary Table S2: Sanger sequencing validation of known mutations

Pt	Sample	Gene	Exon	AA change	Chr	Locus	SNV	VAF (%)
HCC-01	HCC	CNTNAP5	6	R276W*	2	125204422	C/T	28.0
	HGDN_1	IFITM3	1	P70T*	11	320606	G/T	22.2
		MUC4	2	A1646T[r]	3	195513515	C/T	22.5
	HGDN_2	SPTA1	26	R1223Q*	1	158618345	C/T	33.3
		MUC4	2	A1646T[r]	3	195513515	C/T	29.8
	LGDN	SCN11A	15	Q935L*	3	38936055	T/A	20.7
HCC-02	HCC_1	ZNF99	4	E365G	19	22941617	T/C	62.5
	HCC_2	WNK1	7	G612D	12	970393	G/A	21.4
		OGFR	6	G176S*	20	61442874	G/A	20.9
	HGDN	ZNF99	4	E365G	19	22941617	T/C	41.7
	LGDN	MYCBP2	63	S3661L*	13	77657221	G/A	21.0
		OR2T33	1	S87N*	1	248436857	C/T	25.0
		ZFP42	4	R246Q*	4	188924698	G/A	23.3
HCC-03	HCC_2	AAK1	3	I59V*	2	69784099	T/C	22.1
	HGDN_1	FAM47B	1	A534V*	X	34962549	C/T	27.5
		RGL3	5	P207L*	19	11526630	G/A	22.4
HCC-04	HCC	MYO18A	38	R1847C*	17	27414127	G/A	29.1
		PRR16	3	S40F*	5	120021677	C/T	23.3
	LGDN	SOX6	15	G711V*	11	15994629	C/A	36.8
		TDRD6	1	V250M*	6	46656613	G/A	23.8

*Validated by Sanger sequencing, [r] repeat region

Supplementary Table S3: Mutations known in COSMIC or TCGA.

See Supplementary File 2

Supplementary Table S4: Copy number variation regions overlapped with those of liver hepatocellular carcinoma of TCGA (*Brown and green boxes indicate the number of genes located in corresponding regions*).

See Supplementary File 3

Supplementary Table S5: The list of short InDels.

See Supplementary File 4

Supplementary Table S6: HBV virus integrations in HBV-positive cases

Sample ID	Chr	Start	End	Tag count	HBV integrated gene
HCC-02_HCC2	chr3	60163933	60163956	8	FHIT
HCC-03_HCC1	chr1	29128196	29128216	2	
HCC-03_HDN2	chr1	29128196	29128216	2	
HCC-03_HDN1	chr1	148271033	148271124	2	NBPF8, NBPF9, NBPF14
HCC-03_HCC1	chr1	188505529	188505548	2	NBPF8, NBPF9, NBPF14
HCC-03_HCC1	chr10	65900554	65900573	2	
HCC-03_HDN1	chr10	65900554	65900573	2	
HCC-03_HDN2	chr10	65900554	65900573	4	
HCC-03_LGDN	chr10	65900552	65900573	4	
HCC-03_CIR	chr12	5170119	5170139	2	
HCC-03_HDN2	chr13	97789470	97789570	1	
HCC-03_HCC1	chr14	95707983	95708042	1	
HCC-03_HCC1	chr17	1612816	1612836	2	TLCD2
HCC-03_HCC1	chr17	63798729	63798749	2	CEP112
HCC-03_HDN2	chr2	12808508	12808528	2	
HCC-03_HCC1	chr2	223743238	223743257	4	ACSL3
HCC-03_LGDN	chr20	13029597	13029695	2	SPTLC3
HCC-03_HCC1	chr20	47557777	47557900	2	ARFCEF2
HCC-03_HCC1	chr21	11124113	11124278	2	
HCC-03_HCC2	chr3	192192086	192192105	2	FGF12
HCC-03_HDN2	chr5	1295038	1295138	2	TERT
HCC-03_HCC1	chr8	123942201	123942220	2	ZHX2
HCC-03_HCC1	chrX	130169187	130169208	1	
HCC-04_HCC	chr2	12808508	12808528	2	
HCC-04_HDN	chr3	8884249	8884270	2	

Supplementary Table S7: Clinciopathologic chracteristics of four patients with liver transplantation

Pt.		HCC-01	HCC-02	HCC-03	HCC-04
Age		59	58	64	51
Gender		Male	Male	Male	Male
Etiology		HCV	HBV	HBV	HBV
Low grade DN	Number	1	1	1	1
	Size	1cm	0.9cm	1.2cm	0.8cm
High grade DN	Number	3	2	5	2
	Size	up to 1.2cm	up to 1.8cm	up to 2.8cm	up to 1cm
Hepatocellular carcinoma	Number	7	2	2**	5*
	Size	up to 1.2cm	up to 1.7cm	up to 1.1cm	up to 1.2cm
	Grade	II	II	II	I
	Microvessel invasion	No	No	Yes	No
	AJCC stage	2	2	2	2
Background liver		Cirrhosis	Cirrhosis	Cirrhosis	Cirrhosis
Serum Alph-fetoptrotein (ng/mL)		342	20.8	11.1	7.6
Albumin (g/dL)		3	3.4	3	4.4
