# Supplementary material for: Nonlinear tumor evolution from dysplastic nodules to hepatocellular carcinoma
Source: Oncotarget. 2016 Jul 9;8(2):2076–82. doi: 10.18632/oncotarget.10502 (PMC5356781; doi:10.18632/oncotarget.10502)
Supplement: Supplementary file 2 [file oncotarget-08-2076-s002.docx]

**Supplementary Table 1.** Summary of whole exome sequencing.

| Patient | Sample | Nanodrop Con. (ng/ul) | 260/280 | 260/230 | Qubit Con. (ng/ul) | Total amounts (ug) | Real-time PCR (ΔCt) | Total  Reads (M) | Unique Reads (M) | Unique Reads (%) | Unique aligned reads (M) | Unique aligned  reads (%) | Mean Target Coverage |
| --- | --- | --- | --- | --- | --- | --- | --- | --- | --- | --- | --- | --- | --- |
| HCC-01 | CIR | 616.6 | 1.98 | 1.94 | 90.2 | 2.71 | 2.6 | 59.8 | 30.2 | 50.6 | 28 | 92.7 | 35.4 |
|  | LGDN | 810.9 | 2.05 | 1.77 | 78 | 2.34 | 2.3 | 153.8 | 101.9 | 66.3 | 97.2 | 95.3 | 128.6 |
|  | HGDN_1 | 895 | 2.02 | 2.07 | 51.6 | 1.55 | 2.1 | 110.3 | 92.3 | 83.7 | 88.5 | 95.8 | 112.3 |
|  | HGDN_2 | 582.9 | 2.1 | 2.1 | 72.2 | 2.89 | 1.1 | 97.3 | 80.2 | 82.4 | 77.3 | 96.4 | 101.4 |
|  | HCC | 1223 | 2.01 | 2 | 61.2 | 1.84 | 1.9 | 101.6 | 74 | 72.9 | 70.3 | 95 | 85.9 |
| HCC-02 | CIR | 520.2 | 2 | 2.1 | 37.2 | 1.12 | 3.6 | 113.1 | 56.5 | 50 | 53.1 | 94.1 | 73.9 |
|  | LGDN | 413.8 | 2 | 2 | 20.6 | 0.62 | 2.7 | 159.5 | 103.6 | 64.9 | 99.2 | 95.7 | 137.3 |
|  | HGDN | 386 | 2 | 2.2 | 40.6 | 1.22 | 3.4 | 117.3 | 46.4 | 39.5 | 43.5 | 93.7 | 56.7 |
|  | HCC_1 | 328.7 | 2 | 1.6 | 36 | 1.08 | 3.3 | 124.9 | 60.3 | 48.3 | 57 | 94.6 | 78.3 |
|  | HCC_2 | 555.7 | 2 | 2 | 61.6 | 1.85 | 3.2 | 143.3 | 83.8 | 58.5 | 79.7 | 95.1 | 110 |
| HCC-03 | CIR | 462 | 2 | 1.8 | 59.8 | 1.5 | 1.5 | 106.8 | 96.2 | 90.1 | 93.3 | 97 | 128.8 |
|  | LGDN | 531.5 | 2 | 1.8 | 70.2 | 1.76 | 1.7 | 113.6 | 103.5 | 91.1 | 100.2 | 96.8 | 138.8 |
|  | HGDN_1 | 513.2 | 2 | 1.7 | 58.4 | 1.46 | 2.3 | 83.7 | 61.8 | 73.8 | 58.7 | 95 | 67.6 |
|  | HGDN_2 | 794.6 | 2.1 | 2.1 | 86.2 | 2.16 | 1.7 | 102.5 | 90.2 | 88.1 | 87.3 | 96.7 | 120.8 |
|  | HCC_1 | 248.3 | 20 | 1.8 | 42.8 | 1.07 | 1.8 | 105.7 | 94.8 | 89.7 | 91.9 | 96.9 | 126.2 |
|  | HCC_2 | 315.2 | 2 | 2 | 41.8 | 1.05 | 2 | 106.1 | 90.5 | 85.2 | 87.5 | 96.7 | 120.6 |
| HCC-04 | CIR | 838 | 2.1 | 2.1 | 73.4 | 2.2 | 2 | 147.5 | 108.9 | 73.8 | 105.2 | 96.6 | 144.1 |
|  | LGDN | 258.5 | 2.1 | 2.2 | 34.6 | 1.06 | 2.6 | 144.1 | 88.9 | 61.7 | 85.1 | 95.8 | 119.8 |
|  | HGDN | 327.8 | 2 | 2 | 16.8 | 0.5 | 2.1 | 132.4 | 99.7 | 75.3 | 95.6 | 95.9 | 131.8 |
|  | HCC | 546 | 2 | 2 | 89.2 | 2.68 | 2.3 | 134.5 | 81 | 60.2 | 77.7 | 95.8 | 105.6 |
