# Supplementary material for: Nonlinear tumor evolution from dysplastic nodules to hepatocellular carcinoma
Source: Oncotarget. 2016 Jul 9;8(2):2076–82. doi: 10.18632/oncotarget.10502 (PMC5356781; doi:10.18632/oncotarget.10502)
Supplement: Supplementary file 3 [file oncotarget-08-2076-s003.docx]

**Supplementary Table 3.** Mutations known in COSMIC or TCGA.

| ID | Gene | Func | RefseqID | Exon | AAchang | Chr | Start | Ref | Alt | RefC | AltC | VAF | Pvalue | AdjP.FDR |
| --- | --- | --- | --- | --- | --- | --- | --- | --- | --- | --- | --- | --- | --- | --- |
| HCC-01_HCC | *CR1* | synonymous | NM_000573 | 17 | T917T | 1 | 207741317 | C | G | 94 | 19 | 0.168 | 1.43E-21 | 2.15E-19 |
| HCC-01_HCC | *CNTNAP5* | nonsynonymous | NM_130773 | 6 | R276W | 2 | 125204422 | C | T | 72 | 28 | 0.28 | 9.86E-43 | 1.48E-40 |
| HCC-01_HCC | *KLHL24* | nonsynonymous | NM_017644 | 8 | L563I | 3 | 183396958 | C | A | 39 | 7 | 0.152 | 2.98E-09 | 4.47E-07 |
| HCC-01_HCC | *MUC4* | synonymous | NM_018406 | 2 | G1644G | 3 | 195513519 | A | T | 23 | 6 | 0.207 | 2.62E-10 | 3.94E-08 |
| HCC-01_HCC | *ARAP2* | nonsynonymous | NM_015230 | 2 | R57C | 4 | 36230940 | G | A | 18 | 3 | 0.143 | 9.72E-06 | 1.46E-03 |
| HCC-01_HCC | *RNF20* | nonsynonymous | NM_019592 | 16 | R748H | 9 | 104319739 | G | A | 62 | 14 | 0.184 | 7.92E-18 | 1.19E-15 |
| HCC-01_HCC | *DLG5* | synonymous | NM_004747 | 15 | P858P | 10 | 79581668 | T | A | 89 | 31 | 0.258 | 2.58E-38 | 3.87E-36 |
| HCC-01_HCC | *IFITM3* | synonymous | NM_021034 | 1 | P55P | 11 | 320649 | G | A | 15 | 3 | 0.167 | 1.15E-07 | 1.73E-05 |
| HCC-01_HCC | *LUZP2* | synonymous | NM_001252010 | 9 | Q270Q | 11 | 25098952 | G | A | 58 | 14 | 0.194 | 3.83E-17 | 5.75E-15 |
| HCC-01_HCC | *CARS2* | nonsynonymous | NM_024537 | 5 | T160M | 13 | 111340160 | G | A | 40 | 4 | 0.091 | 2.12E-03 | 3.19E-01 |
| HCC-01_HCC | *GAS6* | synonymous | NM_001143946 | 6 | A324A | 13 | 114524944 | A | G | 7 | 9 | 0.563 | 4.48E-14 | 6.72E-12 |
| HCC-01_HCC | *HERC2* | synonymous | NM_004667 | 65 | P3337P | 15 | 28419587 | G | A | 40 | 10 | 0.2 | 1.13E-15 | 1.69E-13 |
| HCC-01_HCC | *CECR1* | nonsynonymous | NM_177405 | 6 | D213Y | 22 | 17662792 | C | A | 20 | 5 | 0.2 | 1.06E-06 | 1.59E-04 |
| HCC-01_HGDN1 | *TAS1R1* | synonymous | NM_138697 | 2 | P122P | 1 | 6631143 | A | T | 77 | 7 | 0.083 | 1.48E-06 | 2.23E-04 |
| HCC-01_HGDN1 | *MUC4* | nonsynonymous | NM_018406 | 2 | S1653I | 3 | 195513493 | C | A | 35 | 5 | 0.125 | 1.62E-02 | 1.00E+00 |
| HCC-01_HGDN1 | *MUC4* | nonsynonymous | NM_018406 | 2 | A1646T | 3 | 195513515 | C | T | 31 | 9 | 0.225 | 1.06E-08 | 1.59E-06 |
| HCC-01_HGDN1 | *MUC4* | synonymous | NM_018406 | 2 | G1644G | 3 | 195513519 | A | T | 31 | 10 | 0.244 | 5.17E-10 | 7.75E-08 |
| HCC-01_HGDN1 | *KIF4B* | synonymous | NM_001099293 | 1 | R742R | 5 | 154395645 | A | T | 296 | 29 | 0.089 | 6.94E-19 | 1.04E-16 |
| HCC-01_HGDN1 | *GARNL3* | synonymous | NM_032293 | 4 | S124S | 9 | 130075772 | C | T | 147 | 16 | 0.098 | 6.43E-18 | 9.64E-16 |
| HCC-01_HGDN1 | *IFITM3* | nonsynonymous | NM_021034 | 1 | P70T | 11 | 320606 | G | T | 21 | 6 | 0.222 | 4.25E-08 | 6.37E-06 |
| HCC-01_HGDN1 | *IFITM3* | synonymous | NM_021034 | 1 | P55P | 11 | 320649 | G | A | 15 | 7 | 0.318 | 1.02E-18 | 1.53E-16 |
| HCC-01_HGDN1 | *TRIM49C* | nonsynonymous | NM_001195234 | 8 | S298N | 11 | 89774252 | G | A | 30 | 4 | 0.118 | 7.54E-04 | 1.13E-01 |
| HCC-01_HGDN1 | *GAS6* | synonymous | NM_001143946 | 6 | A324A | 13 | 114524944 | A | G | 11 | 5 | 0.313 | 2.89E-10 | 4.33E-08 |
| HCC-01_HGDN1 | *HERC2* | synonymous | NM_004667 | 65 | P3337P | 15 | 28419587 | G | A | 42 | 10 | 0.192 | 2.81E-10 | 4.22E-08 |
| HCC-01_HGDN1 | *TBX4* | synonymous | NM_018488 | 3 | Y113Y | 17 | 59543237 | T | C | 199 | 30 | 0.131 | 3.12E-33 | 4.67E-31 |
| HCC-01_HGDN1 | *OR10H1* | synonymous | NM_013940 | 1 | F107F | 19 | 15918527 | G | A | 30 | 7 | 0.189 | 3.39E-06 | 5.08E-04 |
| HCC-01_HGDN1 | *ZNF208* | nonsynonymous | NM_007153 | 4 | S256P | 19 | 22157070 | A | G | 46 | 5 | 0.098 | 2.72E-04 | 4.08E-02 |
| HCC-01_HGDN1 | *ZNF99* | synonymous | NM_001080409 | 4 | G487G | 19 | 22941250 | A | G | 52 | 5 | 0.088 | 2.37E-07 | 3.56E-05 |
| HCC-01_HGDN1 | *ZNF675* | synonymous | NM_138330 | 4 | G346G | 19 | 23836697 | T | G | 39 | 7 | 0.152 | 1.09E-05 | 1.63E-03 |
| HCC-01_HGDN2 | *SPTA1* | nonsynonymous | NM_003126 | 26 | R1223Q | 1 | 158618345 | C | T | 60 | 30 | 0.333 | 3.56E-48 | 5.34E-46 |
| HCC-01_HGDN2 | *MUC4* | synonymous | NM_018406 | 2 | T4203T | 3 | 195505842 | T | G | 11 | 3 | 0.214 | 2.68E-04 | 4.02E-02 |
| HCC-01_HGDN2 | *MUC4* | synonymous | NM_018406 | 2 | T2180T | 3 | 195511911 | G | A | 33 | 4 | 0.108 | 1.54E-02 | 1.00E+00 |
| HCC-01_HGDN2 | *MUC4* | synonymous | NM_018406 | 2 | V1961V | 3 | 195512568 | T | C | 36 | 6 | 0.143 | 1.26E-08 | 1.89E-06 |
| HCC-01_HGDN2 | *MUC4* | nonsynonymous | NM_018406 | 2 | A1646T | 3 | 195513515 | C | T | 33 | 14 | 0.298 | 1.18E-19 | 1.78E-17 |
| HCC-01_HGDN2 | *MUC4* | synonymous | NM_018406 | 2 | G1644G | 3 | 195513519 | A | T | 32 | 13 | 0.289 | 3.54E-20 | 5.31E-18 |
| HCC-01_HGDN2 | *ZNF721* | nonsynonymous | NM_133474 | 3 | V533E | 4 | 436658 | A | T | 66 | 6 | 0.083 | 1.44E-03 | 2.16E-01 |
| HCC-01_HGDN2 | *LCP2* | synonymous | NM_005565 | 16 | V324V | 5 | 169685169 | C | T | 81 | 20 | 0.198 | 8.00E-19 | 1.20E-16 |
| HCC-01_HGDN2 | *OR13C2* | nonsynonymous | NM_001004481 | 1 | Q100L | 9 | 107367610 | T | A | 304 | 32 | 0.095 | 8.84E-25 | 1.33E-22 |
| HCC-01_HGDN2 | *IFITM3* | nonsynonymous | NM_021034 | 1 | P70T | 11 | 320606 | G | T | 32 | 4 | 0.111 | 1.48E-03 | 2.22E-01 |
| HCC-01_HGDN2 | *IFITM3* | synonymous | NM_021034 | 1 | P55P | 11 | 320649 | G | A | 24 | 4 | 0.143 | 6.47E-08 | 9.71E-06 |
| HCC-01_HGDN2 | *GAS6* | synonymous | NM_001143946 | 6 | A324A | 13 | 114524944 | A | G | 8 | 9 | 0.529 | 7.26E-22 | 1.09E-19 |
| HCC-01_HGDN2 | *HERC2* | synonymous | NM_004667 | 65 | P3337P | 15 | 28419587 | G | A | 40 | 13 | 0.245 | 1.21E-11 | 1.81E-09 |
| HCC-01_HGDN2 | *NLGN4X* | synonymous | NM_020742 | 5 | N515N | X | 5821174 | G | A | 26 | 29 | 0.527 | 4.67E-57 | 7.01E-55 |
| HCC-01_LGDN | *LCE1B* | synonymous | NM_178349 | 1 | P21P | 1 | 152784985 | T | A | 174 | 76 | 0.304 | 2.99E-105 | 4.48E-103 |
| HCC-01_LGDN | *ZNF695* | nonsynonymous | NM_020394 | 4 | D439E | 1 | 247150500 | A | T | 64 | 7 | 0.099 | 2.86E-04 | 4.29E-02 |
| HCC-01_LGDN | *SCN11A* | nonsynonymous | NM_014139 | 15 | Q935L | 3 | 38936055 | T | A | 73 | 19 | 0.207 | 1.92E-22 | 2.89E-20 |
| HCC-01_LGDN | *DSPP* | synonymous | NM_014208 | 5 | N1092N | 4 | 88537090 | T | C | 54 | 15 | 0.217 | 1.78E-20 | 2.67E-18 |
| HCC-01_LGDN | *PKHD1L1* | synonymous | NM_177531 | 25 | Y983Y | 8 | 110439334 | C | T | 46 | 6 | 0.115 | 5.92E-05 | 8.88E-03 |
| HCC-01_LGDN | *ADCY8* | nonsynonymous | NM_001115 | 2 | R362C | 8 | 132002665 | G | A | 90 | 18 | 0.167 | 5.65E-25 | 8.48E-23 |
| HCC-01_LGDN | *IFITM3* | synonymous | NM_021034 | 1 | P55P | 11 | 320649 | G | A | 53 | 8 | 0.131 | 5.62E-14 | 8.43E-12 |
| HCC-01_LGDN | *GAS6* | synonymous | NM_001143946 | 6 | A324A | 13 | 114524944 | A | G | 24 | 21 | 0.467 | 4.10E-40 | 6.14E-38 |
| HCC-01_LGDN | *HERC2* | synonymous | NM_004667 | 65 | P3337P | 15 | 28419587 | G | A | 110 | 19 | 0.147 | 9.52E-19 | 1.43E-16 |
| HCC-01_LGDN | *ATXN2L* | nonsynonymous | NM_007245 | 14 | E652Q | 16 | 28844674 | G | C | 134 | 23 | 0.146 | 3.65E-26 | 5.48E-24 |
| HCC-01_LGDN | *ZNF90* | nonsynonymous | NM_007138 | 4 | A534E | 19 | 20229964 | C | A | 35 | 4 | 0.103 | 1.17E-03 | 1.75E-01 |
| HCC-01_LGDN | *ZNF468* | nonsynonymous | NM_001008801 | 4 | S406T | 19 | 53344330 | C | G | 37 | 4 | 0.098 | 8.94E-02 | 1.00E+00 |
| HCC-02_HCC1 | *CFTR* | synonymous | NM_000492 | 10 | N418N | 7 | 117188739 | T | C | 19 | 3 | 0.136 | 2.14E-01 | 1.00E+00 |
| HCC-02_HCC1 | *ANKRD20A2, ANKRD20A3* | synonymous | NM_001012419 | 1 | D45D | 9 | 42368549 | C | T | 24 | 4 | 0.143 | 3.80E-07 | 5.70E-05 |
| HCC-02_HCC1 | *DGKZ* | nonsynonymous | NM_001199266 | 1 | A10D | 11 | 46369238 | C | A | 7 | 4 | 0.364 | 1.14E-04 | 1.71E-02 |
| HCC-02_HCC1 | *AMDHD1* | synonymous | NM_152435 | 6 | I292I | 12 | 96356195 | C | T | 69 | 13 | 0.159 | 6.47E-16 | 9.70E-14 |
| HCC-02_HCC1 | *TPTE2* | nonsynonymous | NM_130785 | 4 | K39E | 13 | 20066994 | T | C | 13 | 3 | 0.188 | 6.33E-05 | 9.50E-03 |
| HCC-02_HCC1 | *KRT38* | stopgain | NM_006771 | 3 | Q235X | 17 | 39595484 | G | A | 37 | 4 | 0.098 | 1.32E-10 | 1.98E-08 |
| HCC-02_HCC1 | *ZNF99* | nonsynonymous | NM_001080409 | 4 | E365G | 19 | 22941617 | T | C | 3 | 5 | 0.625 | 1.23E-13 | 1.85E-11 |
| HCC-02_HCC1 | *MST1* | splicing | NM_020998 | 9 | c.848-2A>C | 3 | 49723916 | T | G | 20 | 3 | 0.13 | 1.69E-04 | 2.53E-02 |
| HCC-02_HCC2 | *OR2T33* | nonsynonymous | NM_001004695 | 1 | S87N | 1 | 248436857 | C | T | 10 | 3 | 0.231 | 1.65E-05 | 2.48E-03 |
| HCC-02_HCC2 | *WNK1* | nonsynonymous | NM_001184985 | 7 | G612D | 12 | 970393 | G | A | 44 | 12 | 0.214 | 6.14E-17 | 9.22E-15 |
| HCC-02_HCC2 | *DNAH9* | stopgain | NM_001372 | 46 | R2947X | 17 | 11725368 | C | T | 24 | 4 | 0.143 | 4.34E-05 | 6.51E-03 |
| HCC-02_HCC2 | *ROCK1* | nonsynonymous | NM_005406 | 31 | Q1217E | 18 | 18534948 | G | C | 36 | 4 | 0.1 | 2.56E-02 | 1.00E+00 |
| HCC-02_HCC2 | *ZNF253* | synonymous | NM_021047 | 4 | P451P | 19 | 20003409 | T | C | 23 | 3 | 0.115 | 1.39E-01 | 1.00E+00 |
| HCC-02_HCC2 | *ZNF99* | nonsynonymous | NM_001080409 | 4 | E365G | 19 | 22941617 | T | C | 5 | 9 | 0.643 | 2.98E-18 | 4.48E-16 |
| HCC-02_HCC2 | *ZNF91* | nonsynonymous | NM_003430 | 4 | K829E | 19 | 23543296 | T | C | 22 | 3 | 0.12 | 2.97E-02 | 1.00E+00 |
| HCC-02_HCC2 | *LILRA6* | nonsynonymous | NM_024318 | 4 | R205Q | 19 | 54745496 | C | T | 43 | 7 | 0.14 | 5.53E-07 | 8.29E-05 |
| HCC-02_HCC2 | *OGFR* | nonsynonymous | NM_007346 | 6 | G176S | 20 | 61442874 | G | A | 178 | 47 | 0.209 | 1.59E-57 | 2.39E-55 |
| HCC-02_HGDN | *TGFBRAP1* | synonymous | NM_001142621 | 4 | I298I | 2 | 105912957 | G | A | 9 | 3 | 0.25 | 6.12E-04 | 9.18E-02 |
| HCC-02_HGDN | *MUC4* | synonymous | NM_018406 | 2 | G1644G | 3 | 195513519 | A | T | 23 | 4 | 0.148 | 2.23E-04 | 3.34E-02 |
| HCC-02_HGDN | *C4orf17* | nonsynonymous | NM_032149 | 7 | A223V | 4 | 100460359 | C | T | 19 | 3 | 0.136 | 9.72E-06 | 1.46E-03 |
| HCC-02_HGDN | *ITIH5* | synonymous | NM_001001851 | 1 | G10G | 10 | 7708826 | C | A | 77 | 31 | 0.287 | 1.10E-52 | 1.65E-50 |
| HCC-02_HGDN | *ZNF99* | nonsynonymous | NM_001080409 | 4 | E365G | 19 | 22941617 | T | C | 14 | 10 | 0.417 | 3.39E-23 | 5.09E-21 |
| HCC-02_HGDN | *MST1* | splicing | NM_020998 | 9 | c.848-2A>C | 3 | 49723916 | T | G | 20 | 3 | 0.13 | 2.91E-05 | 4.37E-03 |
| HCC-02_LGDN | *KCNN3* | synonymous | NM_001204087 | 1 | L66L | 1 | 154842243 | A | C | 61 | 7 | 0.103 | 1.18E-01 | 1.00E+00 |
| HCC-02_LGDN | *OR2T33* | nonsynonymous | NM_001004695 | 1 | S87N | 1 | 248436857 | C | T | 15 | 5 | 0.25 | 1.37E-06 | 2.05E-04 |
| HCC-02_LGDN | *MUC4* | nonsynonymous | NM_018406 | 2 | T4118A | 3 | 195506099 | T | C | 17 | 4 | 0.19 | 1.69E-04 | 2.53E-02 |
| HCC-02_LGDN | *MUC4* | nonsynonymous | NM_018406 | 2 | V3401A | 3 | 195508249 | A | G | 31 | 4 | 0.114 | 6.65E-06 | 9.97E-04 |
| HCC-02_LGDN | *MUC4* | nonsynonymous | NM_018406 | 2 | P2976S | 3 | 195509525 | G | A | 20 | 5 | 0.2 | 5.94E-07 | 8.91E-05 |
| HCC-02_LGDN | *ZFP42* | nonsynonymous | NM_174900 | 4 | R246Q | 4 | 188924698 | G | A | 66 | 20 | 0.233 | 1.68E-23 | 2.52E-21 |
| HCC-02_LGDN | *TUBB* | nonsynonymous | NM_178014 | 4 | E412D | 6 | 30692075 | G | C | 26 | 3 | 0.103 | 3.25E-04 | 4.87E-02 |
| HCC-02_LGDN | *CFTR* | synonymous | NM_000492 | 10 | N418N | 7 | 117188739 | T | C | 34 | 5 | 0.128 | 3.13E-08 | 4.69E-06 |
| HCC-02_LGDN | *ANKRD20A2, ANKRD20A3* | synonymous | NM_001012419 | 1 | D45D | 9 | 42368549 | C | T | 27 | 4 | 0.129 | 1.37E-05 | 2.06E-03 |
| HCC-02_LGDN | *DGKZ* | nonsynonymous | NM_001199266 | 1 | A10D | 11 | 46369238 | C | A | 7 | 4 | 0.364 | 1.55E-04 | 2.33E-02 |
| HCC-02_LGDN | *NUFIP1* | synonymous | NM_012345 | 8 | S372S | 13 | 45523879 | T | C | 24 | 3 | 0.111 | 4.09E-04 | 6.13E-02 |
| HCC-02_LGDN | *MYCBP2* | nonsynonymous | NM_015057 | 63 | S3661L | 13 | 77657221 | G | A | 49 | 13 | 0.21 | 4.19E-21 | 6.29E-19 |
| HCC-02_LGDN | *GOLGA6L1* | synonymous | NM_001001413 | 8 | R540R | 15 | 22743235 | A | G | 12 | 3 | 0.2 | 1.40E-01 | 1.00E+00 |
| HCC-02_LGDN | *KRT38* | stopgain | NM_006771 | 3 | Q235X | 17 | 39595484 | G | A | 89 | 8 | 0.082 | 2.08E-08 | 3.12E-06 |
| HCC-02_LGDN | *ROCK1* | nonsynonymous | NM_005406 | 31 | Q1217E | 18 | 18534948 | G | C | 77 | 10 | 0.115 | 2.61E-04 | 3.91E-02 |
| HCC-02_LGDN | *ZNF99* | nonsynonymous | NM_001080409 | 4 | E365G | 19 | 22941617 | T | C | 10 | 12 | 0.545 | 1.93E-27 | 2.90E-25 |
| HCC-02_LGDN | *SIRPA* | nonsynonymous | NM_001040023 | 2 | G75A | 20 | 1895889 | G | C | 72 | 7 | 0.089 | 5.62E-09 | 8.43E-07 |
| HCC-02_LGDN | *MST1L* | splicing | NM_001271733 | 8 | c.716-2A>C | 1 | 17086183 | T | G | 45 | 4 | 0.082 | 4.65E-03 | 6.97E-01 |
| HCC-03_HCC1 | *MST1L* | synonymous | NM_001271733 | 15 | A670A | 1 | 17083787 | G | A | 37 | 5 | 0.119 | 4.49E-03 | 6.73E-01 |
| HCC-03_HCC1 | *PTCH2* | nonsynonymous | NM_001166292 | 18 | R915C | 1 | 45292393 | G | A | 53 | 6 | 0.102 | 5.14E-06 | 7.72E-04 |
| HCC-03_HCC1 | *NBPF10* | nonsynonymous | NM_001039703 | 6 | E285A | 1 | 145299805 | A | C | 43 | 4 | 0.085 | 3.29E-02 | 1.00E+00 |
| HCC-03_HCC1 | *MUC4* | nonsynonymous | NM_018406 | 2 | T3823I | 3 | 195506983 | G | A | 15 | 3 | 0.167 | 3.01E-03 | 4.51E-01 |
| HCC-03_HCC1 | *SUGT1* | splicing | NM_001130912 | 6 | c.424+2T>C | 13 | 53235707 | T | C | 30 | 3 | 0.091 | 1.17E-03 | 1.75E-01 |
| HCC-03_HCC2 | *MST1L* | synonymous | NM_001271733 | 15 | A670A | 1 | 17083787 | G | A | 25 | 4 | 0.138 | 3.81E-03 | 5.72E-01 |
| HCC-03_HCC2 | *KIAA1522* | nonsynonymous | NM_001198972 | 6 | R451Q | 1 | 33236309 | G | A | 59 | 10 | 0.145 | 8.24E-13 | 1.24E-10 |
| HCC-03_HCC2 | *ADSS* | nonsynonymous | NM_001126 | 1 | E41K | 1 | 244614999 | C | T | 113 | 16 | 0.124 | 1.40E-15 | 2.10E-13 |
| HCC-03_HCC2 | *AAK1* | nonsynonymous | NM_014911 | 3 | I59V | 2 | 69784099 | T | C | 88 | 25 | 0.221 | 4.52E-28 | 6.78E-26 |
| HCC-03_HCC2 | *PRR23A* | nonsynonymous | NM_001134659 | 1 | R111S | 3 | 138724780 | G | T | 143 | 30 | 0.173 | 1.30E-35 | 1.95E-33 |
| HCC-03_HCC2 | *CAPSL* | nonsynonymous | NM_001042625 | 5 | A190T | 5 | 35904706 | C | T | 39 | 6 | 0.133 | 1.22E-05 | 1.84E-03 |
| HCC-03_HCC2 | *HLA-DRB1* | nonsynonymous | NM_002124 | 1 | S24F | 6 | 32557449 | G | A | 9 | 3 | 0.25 | 1.07E-01 | 1.00E+00 |
| HCC-03_HCC2 | *AHNAK2* | nonsynonymous | NM_138420 | 7 | V3209L | 14 | 105412163 | C | G | 40 | 4 | 0.091 | 6.20E-02 | 1.00E+00 |
| HCC-03_HCC2 | *SLC35G6* | nonsynonymous | NM_001102614 | 2 | H59R | 17 | 7385479 | A | G | 22 | 5 | 0.185 | 8.11E-06 | 1.22E-03 |
| HCC-03_HCC2 | *NFE2L1* | nonsynonymous | NM_003204 | 6 | R705C | 17 | 46136797 | C | T | 186 | 41 | 0.181 | 7.27E-42 | 1.09E-39 |
| HCC-03_HGDN1 | *C8orf44* | synonymous | NM_019607 | 2 | A38A | 8 | 67590057 | A | G | 67 | 8 | 0.107 | 3.16E-11 | 4.75E-09 |
| HCC-03_HGDN1 | *C8orf44* | nonsynonymous | NM_019607 | 2 | M42T | 8 | 67590068 | T | C | 48 | 6 | 0.111 | 1.26E-10 | 1.89E-08 |
| HCC-03_HGDN1 | *ZNF682* | synonymous | NM_001077349 | 4 | G221G | 19 | 20117552 | A | T | 65 | 6 | 0.085 | 2.80E-04 | 4.20E-02 |
| HCC-03_HGDN1 | *ZNF737* | synonymous | NM_001159293 | 4 | S308S | 19 | 20728085 | G | A | 91 | 8 | 0.081 | 6.47E-05 | 9.71E-03 |
| HCC-03_HGDN1 | *ZNF99* | nonsynonymous | NM_001080409 | 4 | K618E | 19 | 22940859 | T | C | 88 | 9 | 0.093 | 6.82E-05 | 1.02E-02 |
| HCC-03_HGDN1 | *ZNF701* | nonsynonymous | NM_018260 | 4 | R409P | 19 | 53086538 | G | C | 28 | 4 | 0.125 | 4.75E-03 | 7.12E-01 |
| HCC-03_HGDN1 | *FAM47B* | nonsynonymous | NM_152631 | 1 | A534V | X | 34962549 | C | T | 29 | 11 | 0.275 | 5.15E-20 | 7.73E-18 |
| HCC-03_HGDN1 | *RBMXL3* | nonsynonymous | NM_001145346 | 1 | D398N | X | 114425196 | G | A | 20 | 18 | 0.474 | 8.42E-20 | 1.26E-17 |
| HCC-03_HGDN2 | *ATAD3B* | nonsynonymous | NM_031921 | 16 | P639S | 1 | 1431165 | C | T | 12 | 6 | 0.333 | 4.53E-10 | 6.80E-08 |
| HCC-03_HGDN2 | *NBPF10* | nonsynonymous | NM_001039703 | 6 | E285A | 1 | 145299805 | A | C | 13 | 3 | 0.188 | 2.70E-04 | 4.05E-02 |
| HCC-03_HGDN2 | *CDKN2A* | nonsynonymous | NM_000077 | 2 | H83Y | 9 | 21971111 | G | A | 25 | 4 | 0.138 | 6.99E-05 | 1.05E-02 |
| HCC-03_HGDN2 | *ZNF195* | synonymous | NM_007152 | 4 | K448K | 11 | 3380678 | C | T | 37 | 4 | 0.098 | 9.76E-03 | 1.00E+00 |
| HCC-03_HGDN2 | *LARP4* | nonsynonymous | NM_001170803 | 3 | R80G | 12 | 50822789 | A | G | 17 | 3 | 0.15 | 1.46E-04 | 2.19E-02 |
| HCC-03_HGDN2 | *RGL3* | nonsynonymous | NM_001035223 | 5 | P207L | 19 | 11526630 | G | A | 59 | 17 | 0.224 | 4.19E-21 | 6.29E-19 |
| HCC-03_HGDN2 | *IRGQ* | synonymous | NM_001007561 | 2 | I24I | 19 | 44099419 | G | A | 170 | 24 | 0.124 | 2.79E-22 | 4.18E-20 |
| HCC-03_HGDN2 | *LILRA6* | synonymous | NM_024318 | 5 | R295R | 19 | 54744777 | C | T | 75 | 7 | 0.085 | 2.37E-04 | 3.55E-02 |
| HCC-03_LGDN | *ATAD3B* | nonsynonymous | NM_031921 | 16 | P639S | 1 | 1431165 | C | T | 10 | 4 | 0.286 | 7.35E-09 | 1.10E-06 |
| HCC-03_LGDN | *NBPF10* | nonsynonymous | NM_001039703 | 6 | E285A | 1 | 145299805 | A | C | 33 | 4 | 0.108 | 7.74E-03 | 1.00E+00 |
| HCC-03_LGDN | *GAPVD1* | nonsynonymous | NM_015635 | 21 | P1146S | 9 | 128112629 | C | T | 107 | 14 | 0.116 | 1.66E-18 | 2.49E-16 |
| HCC-03_LGDN | *SLC35G6* | nonsynonymous | NM_001102614 | 2 | H59R | 17 | 7385479 | A | G | 56 | 5 | 0.082 | 4.67E-04 | 7.01E-02 |
| HCC-03_LGDN | *ZNF578* | nonsynonymous | NM_001099694 | 6 | S454P | 19 | 53014994 | T | C | 32 | 4 | 0.111 | 8.58E-03 | 1.00E+00 |
| HCC-04_HCC | *UBE2J2* | synonymous | NM_194457 | 6 | Y199Y | 1 | 1190610 | G | A | 116 | 14 | 0.108 | 4.76E-10 | 7.14E-08 |
| HCC-04_HCC | *LCE1D* | synonymous | NM_178352 | 2 | G68G | 1 | 152770474 | T | A | 39 | 4 | 0.093 | 3.64E-01 | 1.00E+00 |
| HCC-04_HCC | *PRR16* | nonsynonymous | NM_016644 | 3 | S40F | 5 | 120021677 | C | T | 46 | 14 | 0.233 | 4.75E-23 | 7.12E-21 |
| HCC-04_HCC | *EP400* | synonymous | NM_015409 | 47 | Q2726Q | 12 | 132547090 | A | G | 62 | 8 | 0.114 | 1.01E-01 | 1.00E+00 |
| HCC-04_HCC | *MYO18A* | nonsynonymous | NM_078471 | 38 | R1847C | 17 | 27414127 | G | A | 146 | 60 | 0.291 | 1.78E-82 | 2.67E-80 |
| HCC-04_HCC | *ZNF430* | nonsynonymous | NM_001172671 | 5 | N354S | 19 | 21240178 | A | G | 29 | 3 | 0.094 | 7.01E-05 | 1.05E-02 |
| HCC-04_HGDN | *ZNF737* | nonsynonymous | NM_001159293 | 4 | S252T | 19 | 20728254 | C | G | 32 | 3 | 0.086 | 1.08E-04 | 1.62E-02 |
| HCC-04_HGDN | *PSG2* | synonymous | NM_031246 | 2 | T63T | 19 | 43585274 | A | G | 50 | 8 | 0.138 | 2.08E-05 | 3.12E-03 |
| HCC-04_HGDN | *CLIC6* | nonsynonymous | NM_053277 | 1 | S269G | 21 | 36042492 | A | G | 6 | 3 | 0.333 | 4.39E-08 | 6.59E-06 |
| HCC-04_LGDN | *MUC4* | nonsynonymous | NM_018406 | 2 | A4217E | 3 | 195505801 | G | T | 32 | 3 | 0.086 | 4.07E-01 | 1.00E+00 |
| HCC-04_LGDN | *MUC4* | nonsynonymous | NM_018406 | 2 | Q1757H | 3 | 195513180 | C | G | 19 | 3 | 0.136 | 2.72E-03 | 4.08E-01 |
| HCC-04_LGDN | *DAXX* | stopgain | NM_001254717 | 5 | Q476X | 6 | 33287446 | G | A | 48 | 9 | 0.158 | 1.07E-10 | 1.61E-08 |
| HCC-04_LGDN | *TDRD6* | nonsynonymous | NM_001010870 | 1 | V250M | 6 | 46656613 | G | A | 147 | 46 | 0.238 | 9.38E-64 | 1.41E-61 |
| HCC-04_LGDN | *SOX6* | nonsynonymous | NM_001145811 | 15 | G711V | 11 | 15994629 | C | A | 24 | 14 | 0.368 | 3.29E-23 | 4.94E-21 |
| HCC-04_LGDN | *DOC2A* | synonymous | NM_003586 | 2 | G46G | 16 | 30021406 | G | A | 58 | 6 | 0.094 | 2.46E-07 | 3.68E-05 |
| HCC-04_LGDN | *CDC27* | synonymous | NM_001114091 | 6 | T167T | 17 | 45234725 | T | C | 13 | 3 | 0.188 | 8.59E-04 | 1.29E-01 |
| HCC-04_LGDN | *CLIC6* | nonsynonymous | NM_053277 | 1 | S269G | 21 | 36042492 | A | G | 8 | 7 | 0.467 | 1.20E-12 | 1.80E-10 |
| HCC-04_LGDN | *SUPT20HL1* | nonsynonymous | NM_001136234 | 1 | A526P | X | 24382453 | G | C | 30 | 7 | 0.189 | 2.23E-04 | 3.34E-02 |
