# Supplementary material for: Nonlinear tumor evolution from dysplastic nodules to hepatocellular carcinoma
Source: Oncotarget. 2016 Jul 9;8(2):2076–82. doi: 10.18632/oncotarget.10502 (PMC5356781; doi:10.18632/oncotarget.10502)
Supplement: Supplementary file 4 [file oncotarget-08-2076-s004.docx]

**Supplementary Table 4.** Copy number variation regions overlapped with those of liver hepatocellular carcinoma of TCGA (*Brown and green boxes indicate the number of genes located in corresponding regions)*
