# Supplementary material for: Nonlinear tumor evolution from dysplastic nodules to hepatocellular carcinoma
Source: Oncotarget. 2016 Jul 9;8(2):2076–82. doi: 10.18632/oncotarget.10502 (PMC5356781; doi:10.18632/oncotarget.10502)
Supplement: Supplementary file 5 [file oncotarget-08-2076-s005.docx]

**Supplementary Table 5.** The list of short InDels.

| ID | Gene | Func | RefSeq | Chr | position | Exon | NN Change | AA Ghange | RefC | AltC | VAF | Pvalue |
| --- | --- | --- | --- | --- | --- | --- | --- | --- | --- | --- | --- | --- |
| HCC-01_HCC | GGCX | frameshift deletion | NM_001142269 | 2 | 85777144 | 14 | c.2019delA | p.A673fs,GGCX | 77 | 44 | 0.3636 | 2.87E-06 |
| HCC-01_HCC | SETD2 | frameshift deletion | NM_014159 | 3 | 47162729 | 3 | c.3397delC | p.L1133fs | 24 | 8 | 0.25 | 1.96E-02 |
| HCC-01_HCC | ALAS1 | nonframeshift deletion | NM_199166 | 3 | 52236557 | 3 | c.234_254del | p.78_85del,ALAS1 | 103 | 35 | 0.2536 | 5.37E-09 |
| HCC-01_HCC | DSPP | nonframeshift deletion | NM_014208 | 4 | 88536863 | 5 | c.3049_3066del | p.1017_1022del | 54 | 23 | 0.2987 | 6.62E-05 |
| HCC-01_HCC | DSPP | nonframeshift deletion | NM_014208 | 4 | 88537073 | 5 | c.3259_3267del | p.1087_1089del | 38 | 21 | 0.3559 | 1.21E-04 |
| HCC-01_HCC | OR13C2 | frameshift deletion | NM_001004481 | 9 | 107367393 | 1 | c.513_516del | p.171_172del | 87 | 52 | 0.3741 | 5.97E-03 |
| HCC-01_HCC | CTDSP2 | frameshift deletion | NM_005730 | 12 | 58217389 | 8 | c.812delC | p.P271fs | 93 | 25 | 0.2119 | 1.44E-02 |
| HCC-01_HCC | CAND1 | frameshift deletion | NM_018448 | 12 | 67699767 | 10 | c.2319delG | p.M773fs | 61 | 30 | 0.3297 | 1.41E-04 |
| HCC-01_HCC | EP400 | nonframeshift insertion | NM_015409 | 12 | 132547068 | 47 | c.8156_8157insGCA | p.R2719delinsRQ | 17 | 12 | 0.4138 | 1.10E-02 |
| HCC-01_HCC | EPSTI1 | frameshift insertion | NM_001002264 | 13 | 43462434 | 13 | c.1184_1185insCCTGA | p.E395fs | 47 | 31 | 0.3974 | 4.92E-02 |
| HCC-01_HCC | CEACAM19 | frameshift deletion | NM_001127893 | 19 | 45182152 | 4 | c.603delT | p.S201fs,CEACAM19 | 68 | 36 | 0.3462 | 4.28E-06 |
| HCC-01_HCC | TPRX1 | nonframeshift deletion | NM_198479 | 19 | 48305613 | 2 | c.644_655del | p.215_219del | 7 | 12 | 0.6316 | 7.82E-03 |
| HCC-01_HCC | VSIG10L | frameshift deletion | NM_001163922 | 19 | 51845052 | 2 | c.250delG | p.A84fs | 69 | 20 | 0.2247 | 6.08E-03 |
| HCC-01_HCC | NEFH | nonframeshift insertion | NM_021076 | 22 | 29885567 | 4 | c.1938_1939insAAGTCCCCTGAGAAGGCC | p.A646delinsAKSPEKA | 88 | 111 | 0.5578 | 4.14E-13 |
| HCC-01_HCC | RBMXL3 | nonframeshift insertion | NM_001145346 | X | 114425181 | 1 | c.1177_1178insGAGGCCGCTCGCCCAACGCCCACAGCG | p.R393delinsRGRSPNAHSG | 14 | 29 | 0.6744 | 1.94E-09 |
| HCC-01_HGDN1 | DSPP | nonframeshift deletion | NM_014208 | 4 | 88536863 | 5 | c.3049_3066del | p.1017_1022del | 66 | 28 | 0.2979 | 4.83E-05 |
| HCC-01_HGDN1 | DSPP | nonframeshift deletion | NM_014208 | 4 | 88537073 | 5 | c.3259_3267del | p.1087_1089del | 80 | 24 | 0.2308 | 3.64E-03 |
| HCC-01_HGDN1 | OR13C2 | frameshift deletion | NM_001004481 | 9 | 107367393 | 1 | c.513_516del | p.171_172del | 132 | 83 | 0.386 | 3.98E-03 |
| HCC-01_HGDN1 | KRT3 | nonframeshift deletion | NM_057088 | 12 | 53189414 | 1 | c.396_413del | p.132_138del | 36 | 25 | 0.4098 | 1.31E-02 |
| HCC-01_HGDN1 | EPSTI1 | frameshift insertion | NM_001002264 | 13 | 43462434 | 13 | c.1184_1185insCCTGA | p.E395fs | 52 | 48 | 0.48 | 8.41E-03 |
| HCC-01_HGDN1 | KRTAP4-3 | nonframeshift insertion | NM_033187 | 17 | 39324229 | 1 | c.195_196insCTGACCACCTGCTGC | p.R66delinsLTTCCR | 24 | 20 | 0.4545 | 4.69E-03 |
| HCC-01_HGDN1 | NEFH | nonframeshift insertion | NM_021076 | 22 | 29885567 | 4 | c.1938_1939insAAGTCCCCTGAGAAGGCC | p.A646delinsAKSPEKA | 116 | 243 | 0.6769 | 1.31E-21 |
| HCC-01_HGDN2 | PDE4DIP | frameshift deletion | NM_001002811 | 1 | 144923729 | 2 | c.1218delA | p.E406fs,PDE4DIP | 132 | 62 | 0.3196 | 1.09E-02 |
| HCC-01_HGDN2 | HMCN1 | frameshift deletion | NM_031935 | 1 | 185951477 | 18 | c.2746delG | p.G916fs | 83 | 25 | 0.2315 | 1.33E-02 |
| HCC-01_HGDN2 | SETD2 | frameshift insertion | NM_014159 | 3 | 47161803 | 3 | c.4322_4323insG | p.G1441fs | 112 | 33 | 0.2276 | 3.49E-03 |
| HCC-01_HGDN2 | DSPP | nonframeshift deletion | NM_014208 | 4 | 88536863 | 5 | c.3049_3066del | p.1017_1022del | 83 | 45 | 0.3516 | 3.16E-06 |
| HCC-01_HGDN2 | DSPP | nonframeshift deletion | NM_014208 | 4 | 88537073 | 5 | c.3259_3267del | p.1087_1089del | 49 | 14 | 0.2222 | 7.83E-03 |
| HCC-01_HGDN2 | MUC22 | nonframeshift deletion | NM_001198815 | 6 | 30996155 | 3 | c.2947_2976del | p.983_992del | 517 | 140 | 0.2131 | 3.50E-13 |
| HCC-01_HGDN2 | HLA-DRB5 | frameshift insertion | NM_002125 | 6 | 32487158 | 3 | c.640_641insTG | p.T214fs | 207 | 85 | 0.2911 | 9.55E-03 |
| HCC-01_HGDN2 | SMARCA2 | nonframeshift deletion | NM_003070 | 9 | 2039777 | 4 | c.667_669del | p.223_223del,SMARCA2 | 21 | 17 | 0.4474 | 2.66E-02 |
| HCC-01_HGDN2 | MUC6 | nonframeshift deletion | NM_005961 | 11 | 1017496 | 31 | c.5303_5305del | p.1768_1769del | 737 | 222 | 0.2315 | 3.40E-03 |
| HCC-01_HGDN2 | KRT3 | nonframeshift deletion | NM_057088 | 12 | 53189414 | 1 | c.396_413del | p.132_138del | 27 | 19 | 0.413 | 1.71E-02 |
| HCC-01_HGDN2 | CTDSP2 | frameshift deletion | NM_005730 | 12 | 58217389 | 8 | c.812delC | p.P271fs | 115 | 33 | 0.223 | 7.37E-03 |
| HCC-01_HGDN2 | EP400 | nonframeshift insertion | NM_015409 | 12 | 132547068 | 47 | c.8156_8157insGCA | p.R2719delinsRQ | 32 | 32 | 0.5 | 1.72E-04 |
| HCC-01_HGDN2 | EPSTI1 | frameshift insertion | NM_001002264 | 13 | 43462434 | 13 | c.1184_1185insCCTGA | p.E395fs | 50 | 48 | 0.4898 | 6.82E-03 |
| HCC-01_HGDN2 | OR4L1 | frameshift deletion | NM_001004717 | 14 | 20528449 | 1 | c.246_264del | p.82_88del | 296 | 129 | 0.3035 | 3.04E-05 |
| HCC-01_HGDN2 | NEFH | nonframeshift insertion | NM_021076 | 22 | 29885567 | 4 | c.1938_1939insAAGTCCCCTGAGAAGGCC | p.A646delinsAKSPEKA | 96 | 187 | 0.6608 | 1.07E-19 |
| HCC-01_HGDN2 | RBMXL3 | nonframeshift insertion | NM_001145346 | X | 114425181 | 1 | c.1177_1178insGAGGCCGCTCGCCCAACGCCCACAGCG | p.R393delinsRGRSPNAHSG | 10 | 22 | 0.6875 | 7.20E-09 |
| HCC-01_HGDN2 | GPR50 | nonframeshift deletion | NM_004224 | X | 150349558 | 2 | c.1503_1514del | p.501_505del | 24 | 81 | 0.7714 | 5.12E-08 |
| HCC-01_LGDN | DSPP | nonframeshift insertion | NM_014208 | 4 | 88537069 | 5 | c.3255_3256insGATAGCAGC | p.S1085delinsSDSS | 87 | 31 | 0.2627 | 6.99E-05 |
| HCC-01_LGDN | LOC100288524 | frameshift deletion | NM_001195127 | 7 | 331299 | 3 | c.547delC | p.P183fs | 234 | 60 | 0.2041 | 1.70E-04 |
| HCC-01_LGDN | PABPC1 | frameshift insertion | NM_002568 | 8 | 101724623 | 7 | c.938_939insT | p.E313fs | 24 | 8 | 0.25 | 4.88E-02 |
| HCC-01_LGDN | OR13C2 | frameshift deletion | NM_001004481 | 9 | 107367393 | 1 | c.513_516del | p.171_172del | 69 | 29 | 0.2959 | 3.07E-02 |
| HCC-01_LGDN | EP400 | nonframeshift insertion | NM_015409 | 12 | 132547068 | 47 | c.8156_8157insGCA | p.R2719delinsRQ | 82 | 33 | 0.287 | 4.66E-02 |
| HCC-01_LGDN | EPSTI1 | frameshift insertion | NM_001002264 | 13 | 43462434 | 13 | c.1184_1185insCCTGA | p.E395fs | 16 | 17 | 0.5152 | 1.23E-02 |
| HCC-01_LGDN | MESP2 | nonframeshift deletion | NM_001039958 | 15 | 90320121 | 1 | c.533_556del | p.178_186del | 8 | 22 | 0.7333 | 6.25E-04 |
| HCC-01_LGDN | NEFH | nonframeshift insertion | NM_021076 | 22 | 29885567 | 4 | c.1938_1939insAAGTCCCCTGAGAAGGCC | p.A646delinsAKSPEKA | 67 | 147 | 0.6869 | 3.96E-20 |
| HCC-01_LGDN | RBMXL3 | nonframeshift insertion | NM_001145346 | X | 114425181 | 1 | c.1177_1178insGAGGCCGCTCGCCCAACGCCCACAGCG | p.R393delinsRGRSPNAHSG | 26 | 76 | 0.7451 | 2.05E-13 |
| HCC-02_HCC1 | AQP12A | frameshift deletion | NM_198998 | 2 | 241631786 | 2 | c.419delG | p.S140fs | 23 | 12 | 0.3429 | 3.37E-03 |
| HCC-02_HCC1 | ATXN1 | nonframeshift insertion | NM_001128164 | 6 | 16327915 | 7 | c.626_627insGCA | p.H209delinsQH,ATXN1 | 21 | 20 | 0.4878 | 3.41E-03 |
| HCC-02_HCC1 | ZCCHC6 | nonframeshift deletion | NM_001185074 | 9 | 88937852 | 9 | c.2442_2444del | p.814_815del,ZCCHC6 | 4 | 6 | 0.6 | 1.16E-02 |
| HCC-02_HCC1 | NUTM2A | nonframeshift deletion | NM_001099338 | 10 | 88994266 | 7 | c.2438_2440del | p.813_814del | 6 | 7 | 0.5385 | 2.02E-02 |
| HCC-02_HCC1 | NIPA1 | nonframeshift deletion | NM_144599 | 15 | 23086365 | 1 | c.45_47del | p.15_16del | 96 | 40 | 0.2941 | 2.72E-02 |
| HCC-02_HCC1 | KDM6B | nonframeshift deletion | NM_001080424 | 17 | 7751859 | 11 | c.2253_2258del | p.751_753del | 31 | 14 | 0.3111 | 1.78E-02 |
| HCC-02_HCC1 | FADS6 | nonframeshift insertion | NM_178128 | 17 | 72889649 | 1 | c.44_45insTACGGAGCCCATGGAACC | p.P15delinsPTEPMEP | 55 | 100 | 0.6452 | 2.13E-41 |
| HCC-02_HCC1 | RBMXL3 | nonframeshift insertion | NM_001145346 | X | 114425181 | 1 | c.1177_1178insGAGGCCGCTCGCCCAACGCCCACAGCG | p.R393delinsRGRSPNAHSG | 12 | 70 | 0.8537 | 1.07E-29 |
| HCC-02_HCC2 | AFF3 | nonframeshift deletion | NM_001025108 | 2 | 100210335 | 14 | c.1858_1863del | p.620_621del,AFF3 | 29 | 18 | 0.383 | 1.48E-02 |
| HCC-02_HCC2 | ATXN1 | nonframeshift insertion | NM_001128164 | 6 | 16327915 | 7 | c.626_627insGCA | p.H209delinsQH,ATXN1 | 48 | 29 | 0.3766 | 2.61E-02 |
| HCC-02_HCC2 | NIPA1 | nonframeshift deletion | NM_144599 | 15 | 23086365 | 1 | c.45_47del | p.15_16del | 149 | 57 | 0.2767 | 3.77E-02 |
| HCC-02_HCC2 | KDM6B | nonframeshift deletion | NM_001080424 | 17 | 7751859 | 11 | c.2253_2258del | p.751_753del | 78 | 27 | 0.2571 | 3.20E-02 |
| HCC-02_HCC2 | RBMXL3 | nonframeshift insertion | NM_001145346 | X | 114425181 | 1 | c.1177_1178insGAGGCCGCTCGCCCAACGCCCACAGCG | p.R393delinsRGRSPNAHSG | 22 | 98 | 0.8167 | 1.67E-31 |
| HCC-02_HDN | MST1L | frameshift insertion | NM_001271733 | 1 | 17086085 | 7 | c.811_812insG | p.A271fs | 16 | 5 | 0.2381 | 3.39E-02 |
| HCC-02_HDN | ZNF697 | nonframeshift deletion | NM_001080470 | 1 | 120165920 | 3 | c.1026_1046del | p.342_349del | 60 | 18 | 0.2308 | 3.10E-05 |
| HCC-02_HDN | AQP12A | frameshift deletion | NM_198998 | 2 | 241631786 | 2 | c.419delG | p.S140fs | 26 | 9 | 0.2571 | 2.54E-02 |
| HCC-02_HDN | DUX2 | frameshift deletion | NM_012147 | 4 | 190989660 | 1 | c.754delC | p.P252fs | 4 | 5 | 0.5556 | 6.26E-04 |
| HCC-02_HDN | CFTR | frameshift deletion | NM_000492 | 7 | 117188716 | 10 | c.1231_1235del | p.411_412del | 35 | 9 | 0.2045 | 1.93E-03 |
| HCC-02_HDN | FBXO10 | nonframeshift deletion | NM_012166 | 9 | 37537396 | 3 | c.1128_1130del | p.376_377del | 59 | 29 | 0.3295 | 1.85E-15 |
| HCC-02_HDN | ZCCHC6 | nonframeshift deletion | NM_001185074 | 9 | 88937852 | 9 | c.2442_2444del | p.814_815del,ZCCHC6 | 5 | 8 | 0.6154 | 5.58E-03 |
| HCC-02_HDN | OR13C2 | frameshift deletion | NM_001004481 | 9 | 107367665 | 1 | c.243_244del | p.81_82del | 36 | 24 | 0.4 | 2.08E-03 |
| HCC-02_HDN | SH3PXD2A | frameshift deletion | NM_014631 | 10 | 105561048 | 2 | c.145delG | p.D49fs | 10 | 4 | 0.2857 | 1.52E-02 |
| HCC-02_HDN | TBC1D2B | frameshift deletion | NM_015079 | 15 | 78305550 | 9 | c.1885delC | p.Q629fs,TBC1D2B | 74 | 24 | 0.2449 | 4.32E-09 |
| HCC-02_HDN | SNTB2 | frameshift deletion | NM_006750 | 16 | 69221618 | 1 | c.549_555del | p.183_185del | 50 | 17 | 0.2537 | 2.19E-07 |
| HCC-02_HDN | ZNF471 | frameshift deletion | NM_020813 | 19 | 57036769 | 5 | c.1333_1336del | p.445_446del | 8 | 6 | 0.4286 | 7.97E-03 |
| HCC-02_HDN | WNK3 | frameshift deletion | NM_001002838 | X | 54359633 | 2 | c.474delT | p.F158fs,WNK3 | 9 | 3 | 0.25 | 2.41E-02 |
| HCC-02_LGDN | MXRA8 | nonframeshift deletion | NM_032348 | 1 | 1290498 | 5 | c.511_513del | p.171_171del | 87 | 22 | 0.2018 | 6.84E-07 |
| HCC-02_LGDN | MST1L | frameshift deletion | NM_001271733 | 1 | 17087542 | 2 | c.119_123del | p.40_41del | 352 | 102 | 0.2247 | 2.81E-02 |
| HCC-02_LGDN | ZNF697 | nonframeshift deletion | NM_001080470 | 1 | 120165920 | 3 | c.1026_1046del | p.342_349del | 102 | 32 | 0.2388 | 5.74E-06 |
| HCC-02_LGDN | AQP12A | frameshift deletion | NM_198998 | 2 | 241631786 | 2 | c.419delG | p.S140fs | 50 | 13 | 0.2063 | 4.70E-02 |
| HCC-02_LGDN | ZNF717 | frameshift insertion | NM_001128223 | 3 | 75788403 | 5 | c.370_371insA | p.T124fs | 48 | 13 | 0.2131 | 4.74E-02 |
| HCC-02_LGDN | ATXN1 | nonframeshift insertion | NM_001128164 | 6 | 16327915 | 7 | c.626_627insGCA | p.H209delinsQH,ATXN1 | 41 | 26 | 0.3881 | 2.25E-02 |
| HCC-02_LGDN | ANKRD20A3 | frameshift deletion | NM_001012419 | 9 | 43133358 | 1 | c.75delC | p.S25fs,ANKRD20A2 | 8 | 4 | 0.3333 | 4.66E-02 |
| HCC-02_LGDN | ZCCHC6 | nonframeshift deletion | NM_001185074 | 9 | 88937852 | 9 | c.2442_2444del | p.814_815del,ZCCHC6 | 10 | 13 | 0.5652 | 4.13E-03 |
| HCC-02_LGDN | OR13C2 | frameshift deletion | NM_001004481 | 9 | 107367665 | 1 | c.243_244del | p.81_82del | 190 | 76 | 0.2857 | 2.34E-02 |
| HCC-02_LGDN | KRTAP5-1 | nonframeshift deletion | NM_001005922 | 11 | 1606121 | 1 | c.330_359del | p.110_120del | 40 | 17 | 0.2982 | 4.21E-04 |
| HCC-02_LGDN | CDKN1C | nonframeshift deletion | NM_000076 | 11 | 2906197 | 1 | c.512_523del | p.171_175del,CDKN1C | 9 | 11 | 0.55 | 8.23E-03 |
| HCC-02_LGDN | KDM6B | nonframeshift deletion | NM_001080424 | 17 | 7751859 | 11 | c.2253_2258del | p.751_753del | 44 | 20 | 0.3125 | 1.17E-02 |
| HCC-02_LGDN | ABHD17A | nonframeshift deletion | NM_001130111 | 19 | 1881526 | 2 | c.38_40del | p.13_14del,ABHD17A | 12 | 10 | 0.4545 | 4.11E-03 |
| HCC-02_LGDN | RBMXL3 | nonframeshift insertion | NM_001145346 | X | 114425181 | 1 | c.1177_1178insGAGGCCGCTCGCCCAACGCCCACAGCG | p.R393delinsRGRSPNAHSG | 25 | 107 | 0.8106 | 4.63E-32 |
| HCC-03_HCC1 | DSPP | nonframeshift deletion | NM_014208 | 4 | 88536869 | 5 | c.3055_3072del | p.1019_1024del | 17 | 77 | 0.8191 | 4.96E-19 |
| HCC-03_HCC1 | PCDHB12 | frameshift insertion | NM_018932 | 5 | 140590228 | 1 | c.1749_1750insGGTAA | p.G583fs | 16 | 16 | 0.5 | 7.48E-03 |
| HCC-03_HCC1 | LURAP1L | nonframeshift insertion | NM_203403 | 9 | 12775861 | 1 | c.147_148insGGCGGCGGC | p.G49delinsGGGG | 111 | 36 | 0.2449 | 4.12E-02 |
| HCC-03_HCC1 | PABPC3 | frameshift deletion | NM_030979 | 13 | 25671311 | 1 | c.975_979del | p.325_327del | 147 | 49 | 0.25 | 2.54E-02 |
| HCC-03_HCC1 | PABPC3 | frameshift deletion | NM_030979 | 13 | 25671333 | 1 | c.997delA | p.K333fs | 167 | 52 | 0.2374 | 2.98E-02 |
| HCC-03_HCC1 | MESP2 | nonframeshift deletion | NM_001039958 | 15 | 90320121 | 1 | c.533_556del | p.178_186del | 8 | 7 | 0.4667 | 1.86E-02 |
| HCC-03_HCC1 | TPRX1 | nonframeshift deletion | NM_198479 | 19 | 48305667 | 2 | c.578_601del | p.193_201del | 32 | 12 | 0.2727 | 1.38E-04 |
| HCC-03_HCC2 | RPL22 | frameshift insertion | NM_000983 | 1 | 6257784 | 2 | c.44_45insA | p.K15fs | 11 | 5 | 0.3125 | 2.61E-02 |
| HCC-03_HCC2 | ARID1A | frameshift deletion | NM_006015 | 1 | 27106356 | 20 | c.5967delA | p.R1989fs,ARID1A | 130 | 37 | 0.2216 | 3.02E-15 |
| HCC-03_HCC2 | LOR | nonframeshift insertion | NM_000427 | 1 | 153233991 | 2 | c.566_567insCTCTGGCGGCGG | p.Y189delinsYSGGG | 25 | 16 | 0.3902 | 1.35E-02 |
| HCC-03_HCC2 | ZNF717 | frameshift deletion | NM_001128223 | 3 | 75786042 | 5 | c.2732delC | p.S911fs | 12 | 5 | 0.2941 | 4.55E-02 |
| HCC-03_HCC2 | DSPP | nonframeshift insertion | NM_014208 | 4 | 88537069 | 5 | c.3255_3256insGATAGCAGC | p.S1085delinsSDSS | 27 | 45 | 0.625 | 3.71E-20 |
| HCC-03_HCC2 | LURAP1L | nonframeshift insertion | NM_203403 | 9 | 12775861 | 1 | c.147_148insGGCGGCGGC | p.G49delinsGGGG | 103 | 50 | 0.3268 | 8.24E-04 |
| HCC-03_HCC2 | UBTFL1 | frameshift insertion | NM_001143975 | 11 | 89819888 | 1 | c.771_772insT | p.R257fs | 4 | 7 | 0.6364 | 2.14E-02 |
| HCC-03_HCC2 | NIPA1 | nonframeshift deletion | NM_144599 | 15 | 23086365 | 1 | c.45_47del | p.15_16del | 89 | 48 | 0.3504 | 1.77E-02 |
| HCC-03_HCC2 | ONECUT1 | frameshift insertion | NM_004498 | 15 | 53081580 | 1 | c.501_502insC | p.Y168fs | 111 | 31 | 0.2183 | 4.91E-09 |
| HCC-03_HCC2 | MESP2 | nonframeshift deletion | NM_001039958 | 15 | 90320121 | 1 | c.533_556del | p.178_186del | 5 | 15 | 0.75 | 2.00E-04 |
| HCC-03_HDN1 | UBXN11 | nonframeshift deletion | NM_001077262 | 1 | 26608878 | 11 | c.1110_1115del | p.370_372del,UBXN11 | 19 | 11 | 0.3667 | 2.96E-04 |
| HCC-03_HDN1 | TM2D1 | frameshift insertion | NM_032027 | 1 | 62160376 | 5 | c.505_506insT | p.S169fs | 39 | 10 | 0.2041 | 8.61E-03 |
| HCC-03_HDN1 | ALB | frameshift insertion | NM_000477 | 4 | 74279167 | 8 | c.874_875insT | p.Q292fs | 27 | 9 | 0.25 | 2.54E-03 |
| HCC-03_HDN1 | KIAA1244 | frameshift deletion | NM_020340 | 6 | 138629925 | 24 | c.4023delT | p.T1341fs | 43 | 11 | 0.2037 | 6.33E-07 |
| HCC-03_HDN1 | DPY19L1 | frameshift deletion | NM_015283 | 7 | 34981411 | 18 | c.1435_1436del | p.479_479del | 69 | 18 | 0.2069 | 4.43E-02 |
| HCC-03_HDN1 | WNT16 | frameshift insertion | NM_057168 | 7 | 120969360 | 1 | c.14_15insG | p.A5fs | 8 | 2 | 0.2 | 3.27E-02 |
| HCC-03_HDN1 | SFR1 | frameshift deletion | NM_001002759 | 10 | 105881946 | 1 | c.5delC | p.A2fs | 8 | 2 | 0.2 | 4.76E-02 |
| HCC-03_HDN1 | DUX4L7 | frameshift deletion | NM_001127387 | 10 | 135481217 | 1 | c.746delC | p.T249fs,DUX4L5 | 6 | 4 | 0.4 | 5.13E-03 |
| HCC-03_HDN1 | ESRRA | nonframeshift deletion | NM_004451 | 11 | 64083296 | 7 | c.1130_1132del | p.377_378del | 3 | 5 | 0.625 | 1.14E-02 |
| HCC-03_HDN1 | NR4A1 | frameshift insertion | NM_173157 | 12 | 52450855 | 5 | c.1173_1174insC | p.V391fs,NR4A1 | 8 | 2 | 0.2 | 2.54E-02 |
| HCC-03_HDN1 | CTDSP2 | frameshift deletion | NM_005730 | 12 | 58217737 | 7 | c.639_640del | p.213_214del | 132 | 37 | 0.2189 | 4.69E-02 |
| HCC-03_HDN1 | SCAF1 | frameshift deletion | NM_021228 | 19 | 50155036 | 7 | c.1390delG | p.G464fs | 7 | 2 | 0.2222 | 4.18E-02 |
| HCC-03_HDN2 | BUB1 | nonframeshift deletion | NM_004336 | 2 | 111399219 | 21 | c.2623_2625del | p.875_875del | 15 | 4 | 0.2105 | 3.14E-02 |
| HCC-03_HDN2 | PABPC3 | frameshift deletion | NM_030979 | 13 | 25671333 | 1 | c.997delA | p.K333fs | 102 | 34 | 0.25 | 2.90E-02 |
| HCC-03_HDN2 | MESP2 | nonframeshift deletion | NM_001039958 | 15 | 90320121 | 1 | c.533_556del | p.178_186del | 8 | 18 | 0.6923 | 3.44E-04 |
| HCC-03_HDN2 | RBMX | frameshift insertion | NM_002139 | X | 135956571 | 9 | c.905_906insCC | p.P302fs | 18 | 7 | 0.28 | 2.54E-02 |
| HCC-03_LGDN | UBXN11 | nonframeshift deletion | NM_001077262 | 1 | 26608878 | 11 | c.1110_1115del | p.370_372del,UBXN11 | 34 | 24 | 0.4138 | 1.43E-05 |
| HCC-03_LGDN | LOR | nonframeshift insertion | NM_000427 | 1 | 153233991 | 2 | c.566_567insCTCTGGCGGCGG | p.Y189delinsYSGGG | 13 | 10 | 0.4348 | 1.28E-02 |
| HCC-03_LGDN | MIR205HG | nonframeshift deletion | NM_001104548 | 1 | 209605637 | 4 | c.252_263del | p.84_88del | 63 | 31 | 0.3298 | 1.51E-02 |
| HCC-03_LGDN | ASTE1 | frameshift deletion | NM_014065 | 3 | 130733047 | 6 | c.1894delA | p.R632fs | 14 | 6 | 0.3 | 4.76E-03 |
| HCC-03_LGDN | ACVR1B | frameshift insertion | NM_004302 | 12 | 52345593 | 1 | c.66_67insG | p.G22fs,ACVR1B | 63 | 18 | 0.2222 | 1.28E-02 |
| HCC-03_LGDN | MESP2 | nonframeshift deletion | NM_001039958 | 15 | 90320121 | 1 | c.533_556del | p.178_186del | 8 | 11 | 0.5789 | 3.52E-03 |
| HCC-03_LGDN | KRTAP4-3 | nonframeshift insertion | NM_033187 | 17 | 39324229 | 1 | c.195_196insCTGACCACCTGCTGC | p.R66delinsLTTCCR | 78 | 35 | 0.3097 | 3.01E-02 |
| HCC-03_LGDN | RBMX | frameshift insertion | NM_002139 | X | 135956571 | 9 | c.905_906insCC | p.P302fs | 37 | 13 | 0.26 | 1.39E-02 |
| HCC-04_HCC | PLCH2 | nonframeshift insertion | NM_014638 | 1 | 2430086 | 17 | c.2349_2350insGTGGGGGCC | p.E783delinsEVGA | 9 | 6 | 0.4 | 4.61E-02 |
| HCC-04_HCC | KDM1A | frameshift deletion | NM_001009999 | 1 | 23346303 | 1 | c.214delG | p.G72fs,KDM1A | 117 | 37 | 0.2403 | 1.37E-16 |
| HCC-04_HCC | ZNF717 | frameshift insertion | NM_001128223 | 3 | 75786764 | 5 | c.2009_2010insA | p.T670fs | 77 | 34 | 0.3063 | 3.91E-02 |
| HCC-04_HCC | DSPP | nonframeshift deletion | NM_014208 | 4 | 88537073 | 5 | c.3259_3267del | p.1087_1089del | 35 | 17 | 0.3269 | 2.09E-04 |
| HCC-04_HCC | RP1L1 | nonframeshift deletion | NM_178857 | 8 | 10467681 | 4 | c.3922_3927del | p.1308_1309del | 257 | 78 | 0.2328 | 2.45E-02 |
| HCC-04_HCC | KIF21A | frameshift insertion | NM_001173463 | 12 | 39763593 | 3 | c.387_388insA | p.H130fs,KIF21A | 12 | 4 | 0.25 | 3.92E-02 |
| HCC-04_HCC | HBZ | frameshift insertion | NM_005332 | 16 | 203950 | 2 | c.155_156insGTCC | p.G52fs | 59 | 24 | 0.2892 | 1.57E-02 |
| HCC-04_HCC | GRIN3B | nonframeshift deletion | NM_138690 | 19 | 1009551 | 9 | c.3082_3108del | p.1028_1036del | 7 | 5 | 0.4167 | 3.34E-03 |
| HCC-04_HCC | POP4 | frameshift insertion | NM_006627 | 19 | 30106183 | 7 | c.559_560insAAACC | p.E187fs | 23 | 8 | 0.2581 | 1.07E-03 |
| HCC-04_HCC | GPR50 | nonframeshift deletion | NM_004224 | X | 150349558 | 2 | c.1503_1514del | p.501_505del | 36 | 83 | 0.6975 | 1.16E-20 |
| HCC-04_HDN | ZNF717 | frameshift insertion | NM_001128223 | 3 | 75786764 | 5 | c.2009_2010insA | p.T670fs | 76 | 40 | 0.3448 | 8.02E-03 |
| HCC-04_HDN | HTT | nonframeshift deletion | NM_002111 | 4 | 3076604 | 1 | c.52_57del | p.18_19del | 30 | 15 | 0.3333 | 2.12E-02 |
| HCC-04_HDN | TBP | nonframeshift deletion | NM_001172085 | 6 | 170871038 | 2 | c.154_156del | p.52_52del,TBP | 26 | 8 | 0.2353 | 3.81E-02 |
| HCC-04_HDN | FAM90A1 | nonframeshift insertion | NM_018088 | 12 | 8374781 | 7 | c.1031_1032insCGT | p.T344delinsTV | 2 | 28 | 0.9333 | 3.16E-04 |
| HCC-04_HDN | OR4L1 | frameshift deletion | NM_001004717 | 14 | 20528449 | 1 | c.246_264del | p.82_88del | 77 | 168 | 0.6857 | 4.54E-17 |
| HCC-04_HDN | RBMXL3 | nonframeshift insertion | NM_001145346 | X | 114425181 | 1 | c.1177_1178insGAGGCCGCTCGCCCAACGCCCACAGCG | p.R393delinsRGRSPNAHSG | 24 | 62 | 0.7209 | 1.23E-31 |
| HCC-04_HDN | GPR50 | nonframeshift deletion | NM_004224 | X | 150349558 | 2 | c.1503_1514del | p.501_505del | 40 | 124 | 0.7561 | 2.14E-25 |
| HCC-04_LGDN | PLCH2 | nonframeshift insertion | NM_014638 | 1 | 2430086 | 17 | c.2349_2350insGTGGGGGCC | p.E783delinsEVGA | 13 | 11 | 0.4583 | 1.02E-02 |
| HCC-04_LGDN | NOTCH2 | frameshift deletion | NM_001200001 | 1 | 120612003 | 1 | c.17_18del | p.6_6del,NOTCH2 | 107 | 54 | 0.3354 | 1.60E-03 |
| HCC-04_LGDN | PRG4 | nonframeshift deletion | NM_001127710 | 1 | 186276284 | 4 | c.1031_1033del | p.344_345del,PRG4 | 57 | 18 | 0.24 | 1.84E-04 |
| HCC-04_LGDN | ZNF717 | frameshift insertion | NM_001128223 | 3 | 75786764 | 5 | c.2009_2010insA | p.T670fs | 99 | 43 | 0.3028 | 3.46E-02 |
| HCC-04_LGDN | FAM166B | frameshift insertion | NM_001164310 | 9 | 35562057 | 6 | c.774_775insT | p.L259fs | 18 | 5 | 0.2174 | 6.16E-03 |
| HCC-04_LGDN | OR8S1 | frameshift insertion | NM_001005203 | 12 | 48919821 | 1 | c.407_408insGGGTAAAC | p.M136fs | 75 | 24 | 0.2424 | 3.39E-10 |
| HCC-04_LGDN | CTDSP2 | frameshift deletion | NM_005730 | 12 | 58217737 | 7 | c.639_640del | p.213_214del | 318 | 84 | 0.209 | 1.36E-04 |
| HCC-04_LGDN | OR4L1 | frameshift deletion | NM_001004717 | 14 | 20528449 | 1 | c.246_264del | p.82_88del | 57 | 159 | 0.7361 | 5.20E-19 |
| HCC-04_LGDN | HBZ | frameshift insertion | NM_005332 | 16 | 203950 | 2 | c.155_156insGTCC | p.G52fs | 128 | 44 | 0.2558 | 2.27E-02 |
| HCC-04_LGDN | RBMXL3 | nonframeshift insertion | NM_001145346 | X | 114425181 | 1 | c.1177_1178insGAGGCCGCTCGCCCAACGCCCACAGCG | p.R393delinsRGRSPNAHSG | 13 | 62 | 0.8267 | 5.78E-37 |
| HCC-04_LGDN | GPR50 | nonframeshift deletion | NM_004224 | X | 150349558 | 2 | c.1503_1514del | p.501_505del | 37 | 104 | 0.7376 | 1.54E-23 |
